# Spatial localization of β-unsaturated aldehyde markers in murine diabetic kidney tissue by mass spectrometry imaging

**DOI:** 10.1007/s00216-022-04229-7

**Published:** 2022-07-26

**Authors:** Carla Harkin, Karl W. Smith, C. Logan MacKay, Tara Moore, Simon Brockbank, Mark Ruddock, Diego F. Cobice

**Affiliations:** 1 Mass Spectrometry Centre, Biomedical Sciences Research Institute (BMSRI), School of Biomedical Sciences, Ulster University, Coleraine, Northern Ireland UK; 2 National High Magnetic Field Laboratory, Florida State University, Tallahassee, FL 32310-4005 USA; 3 Present Address: Leibniz-Institut für Analytische Wissenschaften—ISAS—e.V., Dortmund, Germany; 4 Scottish Instrumentation and Research Centre for Advanced Mass Spectrometry (SIRCAMS), EastChem School of Chemistry, University of Edinburgh, Edinburgh, Scotland UK; 5 Genomic Medicine, Biomedical Sciences Research Institute (BMSRI), School of Biomedical Sciences, Ulster University, Coleraine, Northern Ireland UK; 6 Randox Laboratories Ltd, 55 The Diamond Rd, Crumlin, UK

**Keywords:** Reactive aldehydes, Mass spectrometry imaging, On-tissue chemical derivatization, Matrix-assisted laser desorption ionization, Diabetic nephropathy

## Abstract

Diabetic nephropathy (DN) is the leading cause of end-stage renal disease. Limitations in current diagnosis and screening methods have sparked a search for more specific and conclusive biomarkers. Hyperglycemic conditions generate a plethora of harmful molecules in circulation and within tissues. Oxidative stress generates reactive α-dicarbonyls and β-unsaturated hydroxyhexenals, which react with proteins to form advanced glycation end products. Mass spectrometry imaging (MSI) enables the detection and spatial localization of molecules in biological tissue sections. Here, for the first time, the localization and semiquantitative analysis of “reactive aldehydes” (RAs) 4-hydroxyhexenal (4-HHE), 4-hydroxynonenal (4-HNE), and 4-oxo-2-nonenal (4-ONE) in the kidney tissues of a diabetic mouse model is presented. Ionization efficiency was enhanced through on-tissue chemical derivatization (OTCD) using Girard’s reagent T (GT), forming positively charged hydrazone derivatives. MSI analysis was performed using matrix-assisted laser desorption ionization (MALDI) coupled with Fourier-transform ion cyclotron resonance mass spectrometry (FT-ICR). RA levels were elevated in diabetic kidney tissues compared to lean controls and localized throughout the kidney sections at a spatial resolution of 100 µm. This was confirmed by liquid extraction surface analysis–MSI (LESA-MSI) and liquid chromatography–mass spectrometry (LC–MS). This method identified β-unsaturated aldehydes as “potential” biomarkers of DN and demonstrated the capability of OTCD-MSI for detection and localization of poorly ionizable molecules by adapting existing chemical derivatization methods. Untargeted exploratory distribution analysis of some precursor lipids was also assessed using MALDI-FT-ICR-MSI.

**Graphical abstract:**

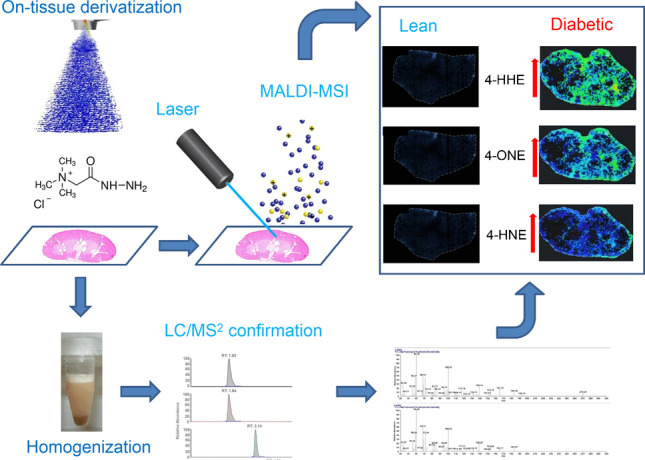

**Supplementary Information:**

The online version contains supplementary material available at 10.1007/s00216-022-04229-7.

## Introduction

The global prevalence of diabetes in adults 20–79 years of age was 463 million in 2019 (9.3% of the global population) [1]. This is projected to rise to 578 million by 2030 (10.2%) and 700 million (10.9%) by 2045 [2]. Diabetic nephropathy (DN) is the leading global cause of end-stage renal disease (ESRD) and is currently diagnosed using a combination of estimated glomerular filtration rate (eGFR) and urinary albumin concentration. Microalbuminuria (30–300 mg L^−1^) signifies a risk of DN development, while macroalbuminuria (> 300 mg L^−1^) signifies overt DN [3]. Non-proteinuric progression of DN to advanced stages [3] and regression of patients with microalbuminuria [4] demonstrate a lack of sensitivity and specificity and highlight a need for alternative biomarkers [5]. An extensive search has been underway in recent years, targeted to reflect biochemical and structural alterations in the nephron prior to albuminuria onset. Markers of a compromised glomerular filtration barrier [6], damaged specialist cells [7, 8], and tubular injury [9–14] have been associated with a renal decline in diabetic patients. In addition, the inflammatory nature of diabetes has led to DN associations with cytokines [15, 16] and growth factors [17, 18].

Oxidative stress leads to the degradation of large biomolecules such as DNA and lipids and modification and deactivation of functional proteins through carbonylation and the formation of advanced glycation end products (AGEs). Small reactive molecules arising from oxidative reactions such as β-unsaturated aldehydes and α-dicarbonyls have been associated with the post-translational modification of proteins in diabetes and DN [19, 20].

Protein carbonylation is an irreversible, non-enzymatic oxidation of proteins by reaction with reactive oxygen species (ROS). This occurs through a primary or direct reaction of the protein with ROS, or through secondary, indirect reactions with by-products of oxidative reactions [17].

Secondary reactions involve the introduction of carbonyl groups by non-covalent adduction. For example, oxidative products of lipid peroxidation act as causative agents of protein carbonylation. In this way, α-β-unsaturated aldehydes (4-hydroxy-trans-2-nonenal (4-HNE), 4-hydroxyhexenal (4-HHE)) and keto-aldehydes (4-oxo-trans-2-nonenal (4-ONE)) also known as “reactive aldehydes” (RAs) produced during lipid peroxidation, react with proteins to form stable Michael adducts [20, 21]. The increased presence of these RAs was found in a diabetic cohort in comparison to controls, and higher again in patients with DN which could be predictive biomarkers for early disease diagnosis and progression [22]. As circulatory levels of RAs can be predictive for the late onset of DN, knowing the intra-tissue kidney generation of these RAs could be predictive of an early onset of the disease and may be potentially used as early-stage biomarkers.

The advent of mass spectrometry imaging (MSI) has unveiled a unique platform with which molecular and spatial information can be obtained simultaneously from thin tissue sections. Matrix-assisted laser desorption ionization (MALDI)-MSI is at the forefront, due to its high spatial resolution and compatibility with biological tissues. Many biomolecules have been imaged in biomedical [21], pharmaceutical [22], forensic [23], and agricultural applications [24]. However, analysis of low molecular weight analytes with poor ionization performance is challenging, particularly, if endogenous levels are low. The application of chemical derivatization has proven successful in alleviating these issues through the addition of highly ionizable moieties. On-tissue chemical derivatization (OTCD) in MSI applications offers an easy method to boost the sensitivity of poorly ionizable molecules on the surface of tissue samples. OTCD has enabled the detection of amines [25], aldehydes/ketones [26, 27], alkenes [28, 29], alcohols [30], thiols [31], and carboxylic acids [32]. Fatty aldehydes (FA) have been previously detected and visualized by Zang and co-workers [33] by hydrogel-assisted derivatization using the Girard P reagent. However, semiquantitative approaches have not been conducted on the specific β-unsaturated hydroxyhexenals by this group.

**Fig. 1:**
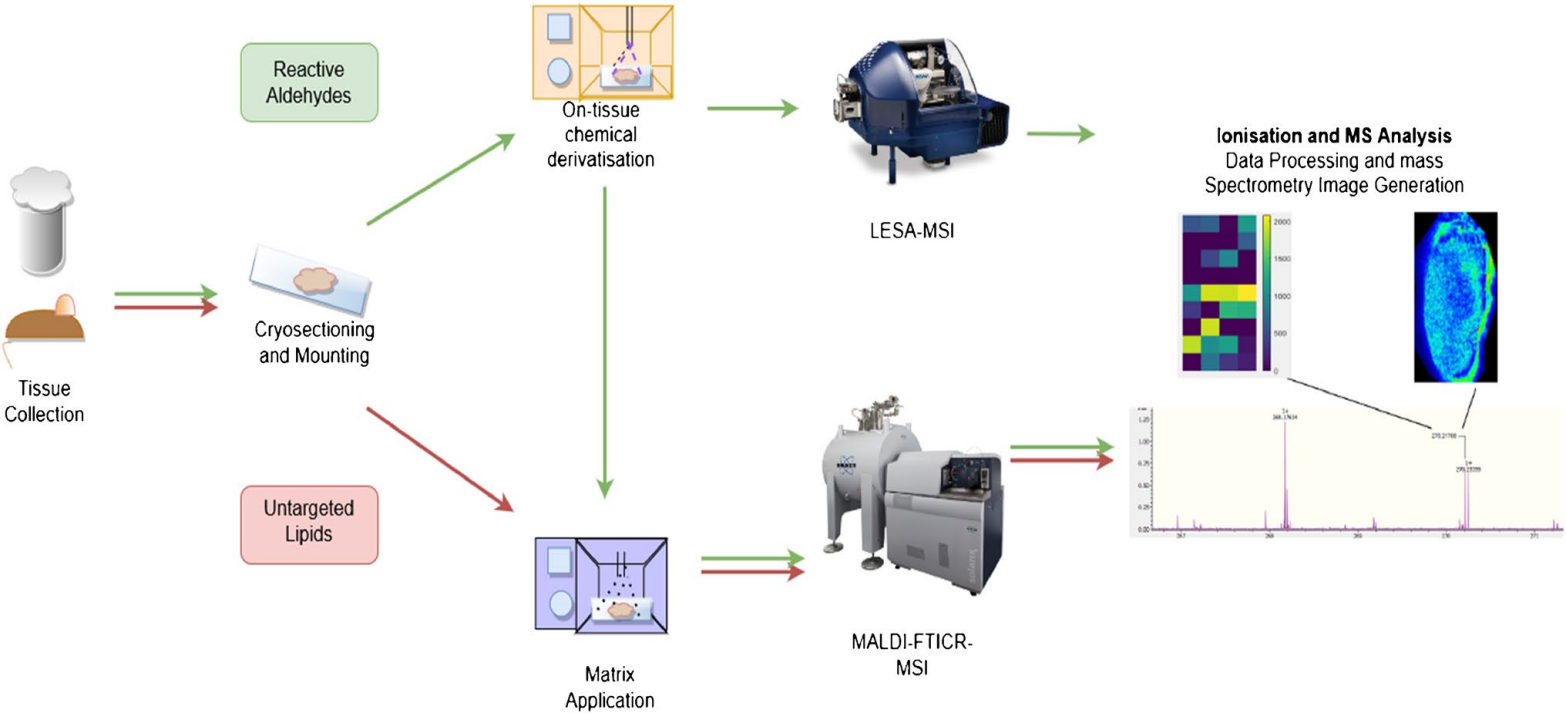
MSI workflow denoting sample preparation for analysis of reactive aldehydes using LESA-MSI and MALDI-FT-ICR-MSI (green arrows) and untargeted lipid analysis using MALDI-FT-ICR-MSI (red arrows)

In this study, the localization and semiquantitative analysis by the incorporation of the internal standard of β-unsaturated hydroxyhexenals 4-hydroxyhexenal (4-HHE), 4-hydroxynonenal (4-HNE), and 4-oxo-2-nonenal (4-ONE) as potential tissue-specific biomarkers for diabetes nephropathy using OTCD-MALDI-FT-ICR-MSI is presented (Fig. 1). For proof-of-concept purposes, this platform was applied to a mouse model of type 2 diabetes (T2DM) using mice with a spontaneous mutation of the leptin receptor (db/db). Specifically, endogenous reactive aldehydes were detected within a mouse kidney using Girard’s reagent T as the derivatization reagent (Supplementary Information, Fig. S1) and results were confirmed by both liquid extraction surface analysis (LESA)-MSI and liquid chromatography tandem mass spectrometry (LC/MS^2^) in tissue homogenate. Untargeted exploratory distribution analysis of some precursor lipids was also assessed using MALDI-FT-ICR-MSI.

## Materials and methods

### Chemicals

Acetone (≥ 99.8%, Honeywell, Arlington, UK), acetonitrile (ACN) (≥ 99.9%, Fisher Scientific, Loughborough, UK), methanol (MeOH) (≥ 99.9%, Honeywell, Arlington, UK, trifluoroacetic acid (TFA, 99%, Sigma-Aldrich, Dorset, UK), and formic acid (~ 98%, Fluka Analytical, Buchs, Switzerland) were HPLC grade/reagent grade. Glyoxal and methylglyoxal (both 40% v/v in water), *p*-anisaldehyde (98% v/v) used as ISTD, and valeraldehyde (pentanal) (≥ 97.5% v/v) were obtained from Sigma-Aldrich, Dorset, UK. RA free serum and tissue homogenate were purchased from Golden West Biological, CA, USA. The remaining aldehyde standards, 4-hydroxyhexenal (4-HHE), 4-hydroxynonenal (4-HNE), and 4-oxo-2-nonenal (4-ONE), were obtained from Cambridge Bioscience (Cambridge, UK). Working stock solutions (1 mg mL^−1^) of aldehyde standards were prepared in ACN and stored at − 20 °C. Girard’s reagent T (GT) (99.0–101.0% w/w), dansyl hydrazine (DH) (≥ 95% w/w), and dinitrophenyl hydrazine (DNPH) were HPLC grade obtained from Sigma-Aldrich (Dorset, UK). MALDI matrix α-cyano-4-hydroxycinnamic acid (CHCA) (≥ 98% w/w) was also obtained from Sigma-Aldrich, (Dorset, UK).

### Animal study and tissue collection

Experiments were conducted in accordance with the Animal (Scientific Procedures) Act 1986. Male mice, eighteen, with a spontaneous mutation of the leptin receptor leading to type 2 diabetes (BKS.Cg- + Lepr^db^/ + Lepr^db^/Ola.Hsd) (db/db) and eighteen lean controls (BKS.Cg-(lean)/Ola.Hsd), 8 weeks old, were purchased from ENVIGO + UK (Bicester, UK) and housed in polypropylene cages in groups of two or three. The temperature was maintained between 20 and 24 °C, and a 12 h light/dark cycle was followed. Standard food and water were provided ad libitum. Six diabetic (db/db) mice and six lean control mice were culled at each of the following time points (TP): 10 weeks, 12 weeks, and 16 weeks of age. Mice were anesthetized by isoflurane injection and a cardiac puncture was performed to acquire a blood sample. Mice were then sacrificed by isoflurane inhalation with confirmation by cervical dislocation. Kidneys were immediately extracted, snap frozen in liquid nitrogen, and stored at − 80 °C until the time of analysis. Blood samples were left to clot for ~ 1 h and the serum supernatant transferred to a clean tube before storage at − 80 °C, prior to analysis by LC/MS^2^.

### Mass spectrometry imaging (MSI)

#### Tissue preparation

Sectioning was performed on one frozen kidney from each mouse using a Leica cryostat (CM 1850 UV; Leica Biosystems, Nußloch, Germany) with water as a mounting medium. Adjacent cross Sects. (12 µm thickness) were taken from a top-down (horizontal) plane and thaw-mounted onto conductive indium tin oxide (ITO)–coated slides (Bruker Daltonik, Bremen, GmBH & CO. KG). Further adjacent Sects. (10 µm thickness) were mounted onto a slide pre-coated with poly-l-lysine for histological staining and a non-coated standard slide for LESA-MSI analysis. The remaining tissue was used for tissue homogenate analysis by LC/MS. All sections were dried in a vacuum desiccator at room temperature for 30 min and stored at − 80 °C for analysis.

#### Matrix-matched standard

A spiked kidney homogenate was prepared using a RA free tissue homogenate (Golden West Biological, CA, USA). The tissue was weighed before transfer to a 1.5-mL centrifuge tube where it was homogenized using a hand-held homogenizer (HMR37M, Herzo, Zhejiang Province, China). Mixed stock solutions of RA standards were prepared and used to spike homogenate with the following concentrations: 4 μg g^−1^, 2 μg g^−1^, 500 ng g^−1^, 100 ng g^−1^, 10 ng g^−1^ tissue; and they were further mixed by ultrasonication. The spiked homogenate was frozen through immersion in a slurry of dry ice/isopropanol and transferred to − 80 °C prior to sectioning.

#### On-tissue chemical derivatization (OTCD)

Tissue sections were removed from − 80 °C and placed into a vacuum desiccator for 30 min at room temperature. Girard’s reagent T (5 mL, 0.1 mg mL^−1^ in 100% MeOH with 0.01% TFA (v/v) was applied using a modified-3D printer (Supplementary Information, Fig. S3) [26, 33] achieving a reagent density of 0.017 mg cm^2−1^. Reaction incubation conditions were adapted as per Cobice et al. [27]. Briefly, tissues were placed in a sealed Petri dish containing moist tissue paper to create a moisturizing reaction environment. Two milliliters of distilled water was enough to produce a suitable reaction media. The moist Kimwipes tissue was placed around the inner walls of the container without touching the glass slide. The tissue was incubated (40 min, 37 °C) in an oven or water bath, then allowed to cool and dry in a vacuum desiccator (RT, 15 min) to remove the condensed water prior to matrix deposition. The ISTD (p-anisaldehyde) was also incorporated onto the reagent at a concentration of 500 ng g^−1^ for semiquantitative purposes. The process took approximately 25 min. Parameters were set as follows: distance from target (*Z*): 23 mm; N_2_ pressure: 1 bar; solvent flow rate: 0.08 mL min^−1^; bed temperature: 30 °C.

#### Matrix application

For MALDI-MSI matrix, CHCA (5 mg mL^−1^) in a 60:40 v/v acetonitrile:water ratio with 0.1% v/v TFA was applied following OTCD using a modified 3D printer (Supplementary Information, Fig. S4) [26, 33]. Parameters were set as follows: distance from target (*Z*): 17 mm; nitrogen pressure: 1 bar; solvent flow rate: 0.11 mL min^−1^; bed temperature: 30 °C. Four passes were required to apply 10 mL of the matrix, achieving an approximate density of 0.25 mg/cm^2^.

#### MALDI-FT-ICR-MSI analysis

Slides were analyzed using a 12 T SolariX Fourier-Transform Ion Cyclotron Resonance Mass Spectrometer (FT-ICR-MS) (Bruker Daltonics MA, US) equipped with a Smartbeam II ™ laser (solid-state 1 kHz laser with advanced optic) MALDI source. The instrument was operated in positive ion mode with a spatial resolution of selected at 100 × 100 µm and 850 shots per pixel. For ion transfer, voltages of funnel 1 and skimmer 1 were adjusted to 150 V and 15 V, respectively. The funnel RF amplitude was adjusted to 150 Vpp. The RF amplitude of the octopole was set to 350 Vpp and its RF frequency to 5 MHz. In the transfer optics section, the time of flight was set to 1.2 ms and the frequency applied to the ICR transfer hexapole rods was set to 4 MHz. The excitation mode was set to sweep mode with a sweep step time of 15 µs. The ramped power excitation was chosen to be continuous (14–28%). The voltages of the ICR Paracell were chosen as follows: transfer exit lens was set to − 20 V and analyzer entrance voltage to − 10 V. Front and back plate voltages were both set to 1.5 V. The side-kick offset was set to − 1.5 V. Under these conditions, a typical mass resolution of ~ 1.5 M FWHM at 400 Da was achieved across the mass range of 150–2000 Da. For high-lateral resolution tissue profiling (H&E staining superimposition), the resolution was set to 45um using a small laser beam and 1500 shots/pixel using the same conditions as mentioned before for whole tissue analysis. Data was processed using Flex Imaging (version 4.1, Bruker Daltonik, Bremen, GmbH & CO. KG) and SCiLS 2019 cPro (Bruker Daltonik, Bremen, GmbH & CO. KG) to obtain images and carry out statistical analysis. All data was normalized by RMS (root mean square) and internally calibrated using both matrix (CHCA) peak at 417.04830 m*/z* and the ISTD (p-anisaldehyde) at *m/z* 266.14991.

For untargeted lipid analysis, the same MALDI-FT-ICR-MS was used as per RA analysis. For MS/MS analysis, quadrupole isolation was checked in the source MS/MS tab. The isolation window was set to 1 m*/z*. Collision cell RF amplitude was adjusted to 2000 V. RF frequency was set to 5. Next, collision-induced dissociation (CID) was selected. The collision energy was increased until the parent ion peak was reduced to 50% of its initial intensity. The remaining parameters were kept as described above. MSiReader (Open source, release 1.02) [34] was used to isolate discriminative peaks between control and diabetic kidney sections and match with a positive ion lipid database (Lipid Maps) https://www.lipidmaps.org/.

#### MALDI-FT-ICR-MSI optimization

Please see Supplementary Information, method 1.3.

##### OTCD-MALDI-FT-ICR-MSI

Based on the results from preliminary and optimization studies, an experiment was designed incorporating kidney tissues from three experimental time points (10 weeks, 12 weeks, and 16 weeks of age) and controls for RA analysis. One kidney from each mouse was cryosectioned for MSI analysis. Kidney Sects. (12 µm) and matrix-matched homogenates at a fixed concentration of 500 ng g^−1^were thaw-mounted onto ITO-coated glass slides (Bruker Daltonik, Bremen, GmbH & CO. KG). Adjacent sections from each diabetic kidney were mounted on three separate slides to analyze each time point (*n* = 6) in the configuration detailed in Supplementary Information, Figure S2b. In total, 54 slides were prepared in this way with 36 analyzed using MALDI-FT-ICR-MSI analysis and 18 with LESA-MSI. For a semiquantitative assessment, RA peak intensities were presented in ratios against the ISTD (RAs/ISTD).

#### Untargeted exploratory lipid analysis by MSI

Please see Supplementary Information, methods 1.4.

#### Confirmatory LESA-MSI analysis

Please see Supplementary Information, methods 1.1 and 1.2.

### Confirmatory LC/MS analysis

To attain further confirmation of the data obtained through MALDI-FT-ICR-MSI and LESA-MSI, RAs were analyzed in homogenates prepared from murine kidney tissue and samples of mouse sera using LC-MS^2^ following solvent extraction and RA derivatization with 2, 4-dinitrophenylhydrazine (DNPH).

#### Standard preparation

Working stock solutions of aldehyde standards (4-HHE, 4-ONE, and 4-HNE) in 100% methanol of 1, 10, 20, 50, 100, and 200 ng g^−1^ for homogenate and 0.5, 1, 5, 25, 50, and 100 ng/m for serum were prepared from working stocks of 0.10 mg mL^−1^.

#### Sample preparation for LC–MS

Kidney tissue homogenates were prepared from shavings retained while cryosectioning for MSI analysis. Methanol and water (400 μL) in a 1:2 w/v ratio were added to 10 mg of kidney shavings followed by an equal volume of ethyl acetate. Samples were incubated on ice for 5 min and ultrasonicated for 10 min to disrupt tissue structure. They were then centrifuged at 18,928 RCF g at 4 °C for 5 min and the upper layer transferred to a clean 1.5-mL tube. A 200 μL volume of the resulting upper layer was further cleaned-up by the addition of 700 μL hexane/acetone (1:1, v/v). The mixture was sonicated for 1 min in a 1.5-mL centrifuge tube and spiked with internal standard (*p*-anisaldehyde, 0.025 ng mL^−1^). Water (MilliQ) (300 μL was added before 10 min sonication and centrifugation at 15,000 g for 10 min. The supernatant was evaporated at RT under N_2_ gas flow. DNPH (100 μL, 0.5 mg mL^−1^ in acetonitrile) was added and samples were left at RT for 2 h. The reaction was quenched with 100 μL water (MilliQ) and transferred to a 1.5 mL HPLC amber vial with an insert. Samples were either analyzed immediately or stored at − 20 °C prior to analysis. For the serum sample, 50 μL of mouse serum was spiked with internal standard (*p*-anisaldehyde, 0.020 ng mL^−1^) and 500 μL of − 20 °C ACN with 0.1% v/v formic acid. The sample was mixed via vortex for 30 s and centrifuged at 18,928 RCF rpm for 5 min to remove protein. The supernatant was transferred to a clean 1.5-mL tube where 700 μL of methyl tert-butyl ether (MTBE) was added before mixing and centrifugation (18,928 RCF for 5 min). A 1 mL volume of the upper layer was evaporated at RT under N_2_ gas flow and reconstituted with derivatization reagent DNPH as described above.

#### ***LC/MS***^***2***^*** conditions***

An Agilent 1250 UHPLC system (Agilent Technologies, Inc, CA, US) coupled to a 6500 Qtrap (ABSciex, LLC, MA, US) was used for RA analysis of both kidney homogenate and mouse serum. Samples and standards were separated on a Luna extended, reverse phase C18 column (50 × 2.1 mm with 3.0 μm particle size) (Phenomenex Inc, CA, USA) at 40 °C, carried by mobile phases (A) 100% acetonitrile and (B) 60:40 v/v water:acetonitrile by gradient elution (Table S2) at a flow rate of 0.40 mL min^−1^. Mobile phases were degassed by ultrasonication for 15 min prior to analysis. Samples were kept at 5 °C and injected in 20 μL volumes (50:50 v/v water:methanol needle wash). The total run time was 35.1 min. MS parameters were set as follows: turbo ion spray source in negative ion mode; dwell time: 150 ms; source temperature: 550 °C; ion source gas 1: 50 psi; ion source gas 2: 50 psi; curtain gas: 30 psi; CAD (collision activated dissociation) gas: 12 psi; ion spray voltage: 5500 V psi. All samples were analyzed in duplicate and linear regression analysis for all standards (ratio against an internal standard) was used to calculate the content of reactive aldehyde in the samples. Data were reported as the average of two injections. For the gradient, see Supplementary Information.

### Histological staining

Please see Supplementary Information, method 1.6.

### Statistical analysis

For MALDI-FT-ICR-MSI analysis, SCiLS 2019 cPro (Bruker Daltonik, Bremen, GmbH & CO. KG) was used for imaging processing and visualization. For MALDI-FT-ICR, LESA-MSI, and LC/MS^2^, statistical analysis was performed using IBM SPSS Statistics for Windows (Version 25) (SPSS, IBM Analytics, New York, USA). Normality of the data distribution was assessed using the Kolmogorov–Smirnov test and through inspection of histograms and Q-Q plots. Normally distributed data followed parametric testing (analysis of variance (ANOVA)) and results were expressed as mean ± SEM whereas data that did not assume normal distribution was analyzed using non-parametric alternatives (Kruskal–Wallis test with post hoc Mann–Whitney *U*) and were expressed as median and interquartile range. Statistical significance was determined from a *p* value of < 0.05. Bland-Alman plots were generated using IBM SPSS Statistics for Windows (Version 25) (SPSS, IBM Analytics, New York, USA) to determine the agreement between MALDI-FT-ICR-MSI and LESA-MSI analysis methods.

## Results

### OTCD-MALDI-FT-ICR-MSI method optimization

In a preliminary experiment following MALDI-FT-ICR-MSI analysis, aldehydes were not detected in dansyl hydrazine (DH)–coated sections; therefore, this reagent was ruled out for further analysis. GT however, facilitated the detection and spatial localization of 4-HHE at *m/z* 228.17035, 4-ONE at *m/z* 268.20169, and 4-HNE at *m/z* 270.21708, observed in both diabetic kidney tissues and lean controls. LESA-MSI analysis following GT-OTCD also demonstrated the increased signal intensity of RAs in diabetic tissues when compared to controls (Supporting Information, Figure S6). Further analysis determined mass accuracies of < 2 ppm for selected RAs using MALDI-FT-ICR-MSI analysis, when compared with their theoretical monoisotopic protonated mass.

### OTCD-MALDI-FT-ICR-MSI study

**Fig. 2:**
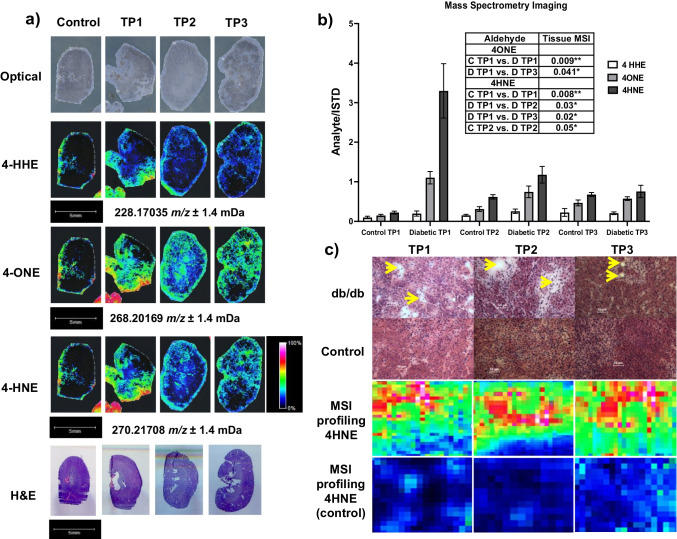
**a** Representative optical, MSI and H&E-stained (adjacent section) images from control (*n* = 6) (left) and diabetic time points (TP) (right) (*n* = 6/per TP). Rows top-down, MSI images obtained for 4-hydroxyhexenal (4-HHE) derivative (*m/z* 228.17035 ± 0.0014 Da), 4-oxo-2-nonenal (4-ONE) derivative (*m/z* 268.20169 ± 0.0014 Da), and 4-hydroxynonenal (4-HNE) derivative (*m/z* 270.21708 ± 0.0014 Da). Data normalized by root mean square (RMS) and calibrated using a CHCA peak (*m/z* 417.04830) and ISTD (p-anisaldehyde) at *m/z* 266.14991. **b** Data obtained from MALDI-FT-ICR-MSI experiment with control and diabetic tissues for RAs: normality of the data distribution was assessed using the Kolmogorov–Smirnov test and through inspection of histograms and Q-Q plots. Normally distributed data followed parametric testing (analysis of variance (ANOVA)) and results were expressed as mean ± SEM. Statistical significance was determined from a *p* value of < 0.05 (*), *p* value of < 0.01 (**), and *p* value of < 0.001 (***). Analysis was performed using IBM SPSS Statistics for Windows (Version 25) (SPSS, IBM Analytics, New York, USA). **c** Hematoxylin and eosin (H&E) stained kidney Sects. (8 µm). Rows: top, diabetic kidney sections (db/db), bottom, control kidney sections. Columns (l-r) TP1 = time point 1, TP2 = time point 2, TP3 = time point 3. Yellow arrows indicate possible carbonylated by-product deposition observed in diabetic tissues. Bottom, panel, MSI images of 4HNE at higher spatial resolution (45 μm)

**Fig. 3:**
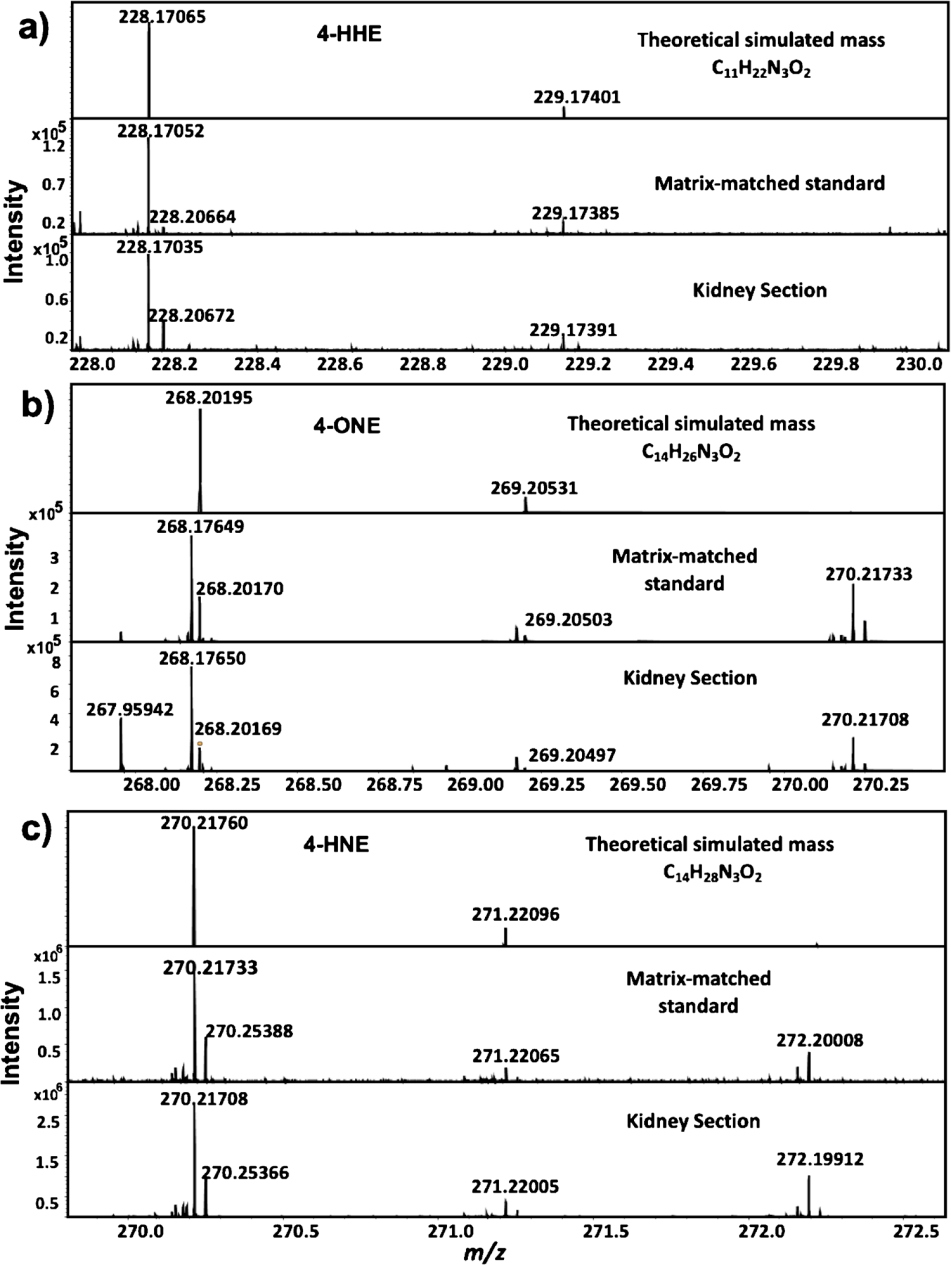
Data obtained from representative MALDI-FT-ICR-MSI experiment with control and diabetic tissues for RAs: **a** 4-HHE, **b** 4-ONE, and **c** 4-HNE. Spectral overlay of theoretical simulated peaks with those of matrix-matched standard and kidney sections (data normalized by root mean square and calibrated using a CHCA peak (*m/z* 417.04830) and the ISTD (p-anisaldehyde) at *m/z* 266.14991 Scale and intensity bars inset)

Figure 2a displays the results of a representative analysis of three slides using MALDI-FT-ICR-MSI. In general, an increase in the signal intensity of RAs was observed in diabetic tissue sections in comparison to control tissues except for time point 3 (TP3). As shown in Table 1, all RA derivatives displayed a mass accuracy of < 3 ppm within matrix-matched standards and kidney sections compared with their corresponding theoretical simulated protonated masses. Representative RA derivatives FT-ICR spectra of tissue, matrix-matched standards are shown in Fig. 3. Kruskal–Wallis with post hoc Wilcoxon rank-sum statistical analysis was employed to assess differences in the mean spectra of RA *m/z* intervals between ROIs (ROIs = whole tissue). As shown in Fig. 2b, the most significant increase was observed at TP1 for both 4-HNE (*p* = 0.008) and 4ONE (*p* = 0.008); 4-HNE was also found to be significant at TP2 but less than TP1 which is a promising demonstration of the potential utility of these RA as early DN tissue biomarkers. Then, all RAs were tailing off to control levels. No significant changes were observed for 4-HHE. Regarding localization (Fig. 2a), the distribution of RAs was scattered across the entire kidney section; however, the signal intensity was mainly detected at the cortex for 4-HHE, whereas for 4-ONE and 4-HNE, it was observed around the medulla.

**Table 1:** Mass accuracies (ppm) obtained from representative slides analyzed using MALDI-FT-ICR-MSI for the matrix-matched standard and the kidney sections

			Matrix-matched standard		Kidney section	
Analyte	Theoretical *m/z*	Slide no	*m/z*	Mass error (ppm)	*m/z*	Mass error (ppm)
4-HHE	228.17065	C	228.17035	1.31	228.17052	0.57
		TP1	228.17018	2.06	228.17022	1.88
		TP2	228.17052	0.57	228.17035	1.31
		TP3	228.17022	1.88	228.17024	1.80
4-ONE	268.20195	C	268.20147	1.79	268.20171	0.89
		TP1	268.20173	0.82	268.20194	0.04
		TP2	268.20182	0.48	268.20178	0.63
		TP3	268.20171	0.89	268.20180	0.56
4-HNE	270.21760	C	270.21732	1.04	270.21737	0.85
		TP2	270.21732	1.04	270.21731	1.07
		TP2	270.21732	1.04	270.21755	0.19
		TP3	270.21726	1.26	270.21728	1.18

As displayed in Fig. 2c, histological assessment demonstrated white lesions within diabetic kidney sections around the medulla imaged at × 20 magnification. The presence of white lesions, indicated by yellow arrows, was increased in the diabetic tissue, and showed molecular co-localization with the distribution of 4-HNE obtained at the same area in profiling mode at a higher lateral resolution. Only 4-HNE was monitored in this experiment since it was the most relevant marker for the whole tissue MSI experiments.

Regarding methodological verification using an orthogonal technique, Bland–Altman plots demonstrate the agreement between LESA-MSI and MALDI-FT-ICR-MSI methods for RA analysis in kidney tissues (Supplementary Information, Figure S8). LESA-MSI MRM data was normalized to be comparable with the data from the FT-ICR-MSI analysis. There does not seem to be a trend with increased data points above or below the mean difference line, suggesting that there is no bias in the measurements. Panel (B) shows a linear regression model; the *R*^2^ value is indicated as > 0.9, suggesting good agreement between both methods. Further analyte identification was conducted by comparing RAs on-tissue product ion spectra against pure standards (Supplementary Information, Figure S10–12).

### Confirmatory LC/MS analysis

**Fig. 4:**
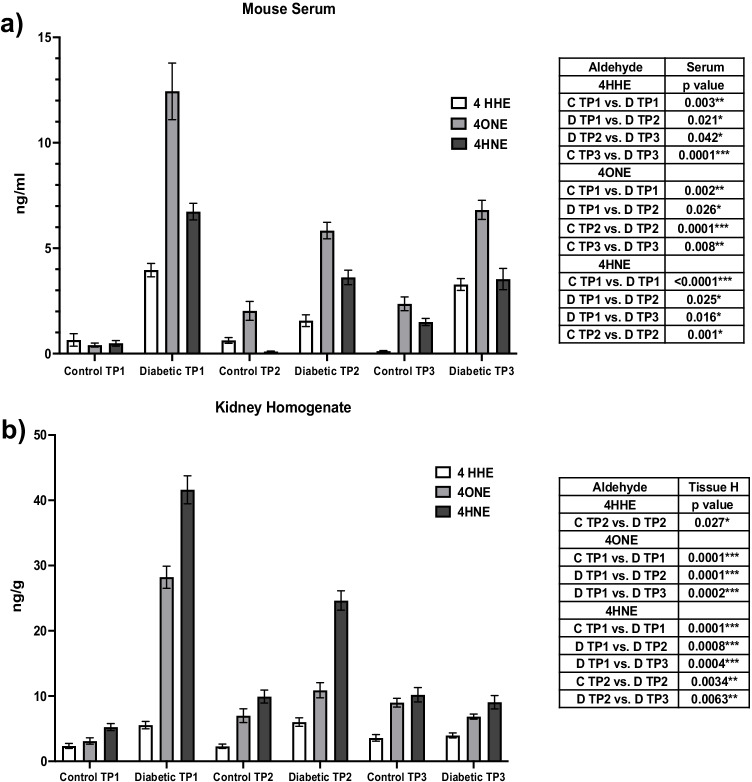
Concentrations of RAs (ng/mL) in serum samples **a** and **b** kidney homogenate samples for control and diabetic mice at three time points (TP) *n* = 6/per TP. Normality of the data distribution was assessed using the Kolmogorov–Smirnov test and through inspection of histograms and Q-Q plots. Normally distributed data followed parametric testing (analysis of variance (ANOVA)) and results were expressed as mean ± SEM. Statistical significance was determined from a *p* value of < 0.05 (*), *p* value of < 0.01 (**), and *p* value of < 0.001 (***). Analysis was performed using IBM SPSS Statistics for Windows (Version 25) (SPSS, IBM Analytics, New York, USA

RAs in mouse serum and kidney tissue homogenate samples were analyzed by LC/MS^2^ following solvent extraction and derivatization with 2,4-DNPH. As shown in Fig. 4a, in serum, 4-HHE was increased at TPI vs control (*p* = 0.003), then decreased at TP2 vs TPI (*p* = 0.021) showing a further increase at TP3 from TP2 (*p* = 0.042). On the contrary, 4-ONE displayed the highest increase at TPI vs control (*p* = 0.002) with the same behavior observed as 4-HHE at TP2 (*p* = 0.026) with the exception that no significant differences between TP2 and TP3 were observed. Finally, 4-HNE displayed the same pattern as ONE with a very significant elevation at TPI vs control (*p* < 0.0001) following a subsequent decrease at TP2 vs TPI (*p* = 0.025) and finally reaching a plateau at TP3 from TP2.

In contrast to serum, tissue homogenate levels of RAs (Fig. 4b) presented a sharp increase at TPI followed by a sustainable decline for TP2 and TP3 except for 4-HHE which was only significant at TP2 vs control. Levels of 4-ONE were sharply increased for TP1 against control (*p* = 0.0001) and declined at TP2 vs TP1 (*p* = 0.0001) and then remained constant at TP3. Regarding 4HNE, levels were climbed at TP1 vs control (*p* = 0.0001) and then tailed off at both TP2 vs TPI (*p* = 0.0008) and TP3 vs TP2 (*p* = 0.0063). However, as found in all RAs, no significant differences were observed in TP3 vs control.

These findings support the elevation in RA concentration in the early onset of DN. A comparison table with serum, tissue homogenate, and MSI data can be found in Table 2.

**Table 2:** *p* values for RAs detected in mouse serum, kidney homogenate, and MSI. *p* value obtained through Mann–Whitney *U* comparison of control vs diabetic tissues for each time point using IBM Statistics for Windows (Version 25) (SPSS, IBM Analytics, New York, USA)

Aldehyde	Serum	Tissue H	Tissue MSI
	***p* value**		
**4-HHE**			
C TP1 vs. D TP1	0.003**	0.052	0.787
D TP1 vs. D TP2	0.021*	0.997	0.908
D TP1 vs. D TP3	0.13	0.163	0.999
C TP2 vs. D TP2	0.128	0.027*	0.387
D TP2 vs. D TP3	0.042*	0.111	0.988
C TP3 vs. D TP3	0.0001***	0.971	0.977
**4-ONE**			
C TP1 vs. D TP1	0.002**	0.0001***	0.009**
D TP1 vs. D TP2	0.026*	0.0001***	0.154
D TP1 vs. D TP3	0.069	0.0002***	0.041*
C TP2 vs. D TP2	0.0001***	0.491	0.344
D TP2 vs. D TP3	0.726	0.061	0.487
C TP3 vs. D TP3	0.008**	0.110	0.241
**4-HNE**			
C TP1 vs. D TP1	< 0.0001***	0.0001***	0.04*
D TP1 vs. D TP2	0.025*	0.0008***	0.03*
D TP1 vs. D TP3	0.016*	0.0004***	0.02*
C TP2 vs. D TP2	0.001*	0.0034**	0.05*
D TP2 vs. D TP3	> 0.999	0.0063**	0.292
C TP3 vs. D TP3	0.079	0.961	0.981

### Exploratory screening lipid MSI analysis

An exploratory lipid analysis by MSI was conducted on kidney sections to investigate the differences between lipid profiles of diabetic and control tissues. MSiReader (Open source Release 1.02) [35] identified discriminatory lipid peaks (using Lipid Maps database, https://www.lipidmaps.org) between control (*n* = 3) and diabetic tissues (*n* = 3/TP) which were matched against a positive ion database with < 5 ppm mass accuracy. FlexImaging was then used to visualize the matched lipid *m/z* intervals. Positive ion-matched peaks are listed in Table S3. The signal for all these lipids was increased in diabetic tissues as observed in the MS images, except at *m/z* 732.55378 [M + H]^+^ (PC (18:1_14:0)) which was higher in the control tissue. At TP1, ions at *m/z* 703.57485 [M + H]^+^ (SM (18:1_16:0)) and 732.55378 [M + H]^+^ (PC (18:1_14:0)) were spatially distributed at a higher intensity towards the cortex, while ions of *m/z* 723.49352 [M + Na]^+^ (PA (18:0_18:2) and 798.54081 [M + K]^+^ (PC (18:1_16:0)) were at a higher intensity towards the medulla. At TP2 and TP3, all ion intensities were spatially distributed at a higher intensity in the medulla, except for *m/z* 732.55378 which is higher in the cortex. Lipid species were further confirmed by tandem MS/MS analysis using Lipid Blast version Full-Release-3, https://doi.org/10.1038/nmeth.2551 (Fiehn Lab, CA, US). The discrimination between control and diabetic tissues demonstrates a change in lipid profile in DN and the possible relationship between lipid peroxidation and RA generation.

## Discussion

In this study, an OTCD-MALDI-MSI method was adapted and applied for the detection and visualization of low molecular weight reactive aldehydes in kidney sections of diabetic mice and aimed to identify their potential as possible biomarkers of diabetic nephropathy (DN). β-unsaturated aldehydes and α-dicarbonyls are precursors of the advanced glycation end product (AGE) formation and levels have been shown to be elevated in diabetic and DN patients [19, 36]. Aldehyde groups react with amino acid side chains of proteins to produce Schiff bases. Further rearrangement and cross-linking results in stable, irreversible AGEs, which carry carbonylated moieties. MALDI-FT-ICR-MSI analysis of kidney tissues identified derivatized RAs and demonstrated elevated levels in diabetic tissues. This finding was supported by LESA-MSI and LC/MS^2^ analysis of kidney homogenate and serum.

A mouse model of spontaneous type 2 diabetes was chosen for this study which exhibits histological changes including glomerular basement membrane thickening, podocyte reduction, and mesangial matrix expansion, which signify its usefulness in investigating the early stages of DN. In addition, studies have also reported oxidative stress and inflammation in this model [37, 38].

The FT-ICR analyzer was selected due to its unparalleled mass resolution, critical for the analysis of complex biological matrices and confident molecule identification. RA mass accuracies of < 3 ppm were attained within kidney sections and matrix-matched standards when compared with simulated theoretical peaks and spatial resolution of 100 μm was achieved allowing analyte localization within kidney sections. An OTCD method was adapted in which mounted tissue sections were coated with a derivatization reagent using a modified 3D printer. This method has previously proven effective for the application of derivatization and MALDI matrices to tissues prior to MALDI-MSI experimentation [26, 33].

Derivatization reagent screening to target carbonyl moieties using MALDI-MSI was carried out using hydrazone-forming reagents (GT and DH) with GT previously identified as more effective in OTCD methods targeting carbonyl moieties of steroid molecules [22, 27, 33]. GT reacts with aldehydes in a two-step condensation reaction normally achieved by using a protic solvent in a weak acid media as a catalyst. It contains a positively charged trimethylamine and a reactive acetyl hydrazine, which forms stable hydrazones with carbonyl groups (Supporting Information, Figure S1). 4-HHE, 4-ONE, and 4-HNE GT were successfully detected as hydrazone derivatives in a preliminary study. Dansyl hydrazine (DH) was also screened alongside GT. However, DH did not produce any aldehyde derivative signals in this experiment. In DH derivatization for LC–MS, high temperatures [39] and long incubation times [40, 41] are required for carbonyl derivatization. It is possible that the mild OTCD conditions were not suitable to produce good reaction yields. No further reagents were evaluated as previously published studies for the detection of steroids found GT as the best reagent for MALDI-MSI targeting carbonyl moieties. This reagent has been used to map carbonyl-containing molecules in a plethora of biological tissues [22, 26, 27, 33, 42–45]. In this study, optimized conditions were used as previously reported [27, 33]; therefore, no additional reaction condition assessment was necessary. α-cyano-4-hydroxycinnamic acid was used as the MALDI matrix as previously reported for GT-derivatives using the same MALDI-MSI platform [27]. No isobaric contributions were observed from both matrix clusters and endogenous metabolites. A representative total ion chromatogram (TIC) is shown in Supplementary Information, Figure S9.

Regarding sample preparation, some tissue damage and analyte delocalization were observed, particularly, in the diabetics’ mouse tissue. The damage observed could be mainly associated with the combination of cryosectioning and on-tissue chemical derivatization. These genetically modified obese diabetic mice presented with substantial adipose deposits across the kidney tissue at the time of culling. Since cryosectioning was performed at − 20 °C, fat deposits may have caused damage to tissue during sectioning as fat was in its quasi-semisolid state at the cryosectioning temperature. Moreover, the co-solvent used in the OTCD reaction may have dissolved the fat deposits across the tissue section causing undesired morphological changes and analyte delocalization. A possible way to mitigate or reduce this issue could be to lower the cryosection temperature, e.g., 30 °C, and recued the incubation time of the OTCD reaction.

Experimental time points were designed to reflect changes in the weeks shortly following hyperglycemia onset, the beginning of the renal decline, and a mid-point. Analysis revealed significant differences between sections in all slides tested, suggesting a basis for further investigation into RAs as possible indicators of DN onset and progression in diabetes.

As shown in Fig. 2b, levels of 4-HNE and 4-ONE peaked at T1 (onset of hyperglycemia time point) and tailed off at T2 and T3. 4-HNE is the physiological most abundant RA formed as a by-product of lipid peroxidation and 4-ONE is an oxidative degradation product of 4-HNE formed by oxidation of the ketone moiety at position C4. We hypothesized that the RAs are reduced in the kidney as they are incorporated into proteins and other macromolecules in the tissues through protein carbonylation and the formation of advanced glycation end products. Alternatively, with the progression of DN, and nephron loss, reduced renal blood flow may reduce RAs making their way through the glomerulus to the kidney tissues. This data suggests that they all would make potential biomarkers for T2DM and confirms the knowledge that an oxidative diabetic environment is induced.

In addition, the suitability of the OTCD-MSI method for RA detection and localization evaluation within tissue sections was clearly demonstrated. MSI techniques are increasing in popularity due to improvements in spatial resolution and sample preparation methodologies allowing better sample-sample reproductivity, less analyte delocalization, and, therefore, more accurate results [46–48]. LESA-MSI coupled to a triple quadrupole analyzer was used for cross-validation purposes as an orthogonal MSI approach. Since LESA spatial resolution was not comparable to MALDI, the assessment was only focused on RA signal intensity between the studied groups, which followed the same trend as observed using MALDI-MSI.

The histological assessment demonstrated white lesions within diabetic kidney sections. These could be attributable to carbonylated-related by-products resulting from ectopic lipoprotein or protein accumulation, which has been associated with DN and insulin resistance [49, 50]. This finding correlates with the increase of 4 HNE observed in diabetic kidney tissues through MALDI-FT-ICR-MSI profiling analysis [51].

An analysis of tissue homogenate samples by LC-MS^2^ also supported the MSI data, with higher concentrations of RA observed in diabetic tissues than in controls. In addition, serum levels were also correlated with levels found in tissue homogenate, suggesting an active tissue-circulation transport mechanism. This may offer the possibility to use a less invasive screening diagnostic procedure through blood testing, a more efficient and less invasive diagnostic procedure than that of a tissue biopsy.

MALDI-FT-ICR-MSI analysis of untargeted lipids displayed alterations in certain lipid species within diabetic tissues. There have been many studies which suggest that lipid imbalance and accumulation in non-adipose tissues impair insulin signaling and contribute to the alteration of organ function [51–54]. Phosphatidylcholine (PC) and sphingomyelin (SM) chain length have been shown to alter their function in relation to insulin resistance: longer carbon-chained molecules demonstrate a protective function; however, shorter chained molecules seem to have the opposite effect [52]. This extends to renal protection, with longer PC and SM chains associated with a reduced risk of renal impairment [55]. SM levels within glomeruli are increased in the diabetic kidney and are believed to fuel an increase in ATP levels, suppressing AMP-activated protein kinase (AMPK), resulting in mitochondrial dysfunction and contributing to renal injury [56, 57]. In addition, bioactive lysophosphatidlycholine (LPC) species were found to be increased in diabetic kidneys, spatially localized to the glomeruli and more prominently in those which have a higher degree of renal fibrosis [57, 58].

Detection and localization of RAs in renal tissue was the first step in gaining an understanding of RA changes in kidney tissues of diabetic mice, and therefore suggesting a correlation with DN progression. Further investigation into the alterations in lipid profiles may provide a connection from the peroxidation of lipid species to RA generation in renal tissues. Furthermore, elevated RAs in serum exhibited an adaptable platform by which RAs could be measured in the serum of diabetic patients in a clinical setting [59–63].

## Conclusion

In this study, we have shown that the combination of MALDI-FT-ICR-MSI with OTCD is a powerful new tool for the detection and semi-quantitation of small reactive aldehydes in mouse kidney tissue sections. We have demonstrated its utility for measuring endogenous concentrations within a mouse model of type 2 diabetes (T2DM) using mice with a spontaneous mutation of the leptin receptor (db/db). This offers the opportunity for many novel insights into the tissue-specific mechanism of diabetic nephropathy. In addition, oxidative stress is not only restricted to kidney tissues in diabetes and so there is a possibility of the application of this technique to other tissues, such as retinal neurovascular models in the investigation of diabetic retinopathy. In summary, the presented OTCD-MALDI-MSI method facilitated the analysis of small reactive aldehydes in diabetic kidney tissues and demonstrates their utility as “potential” biomarkers of disease diagnosis and progression.

## Supplementary Information

Below is the link to the electronic supplementary material.

Supplementary file1 (PDF 1.88 mb)

## Abbreviations

MSI: Mass spectrometry imaging
LC/MS: Liquid chromatography tandem mass spectrometry
LESA: Liquid extraction surface analysis
OTCD: On-tissue chemical derivatization
4-HHE: 4-Hydroxyhexenal
4-HNE: 4-Hydroxynonenal
4-ONE: 4-Oxo-2-nonenal
