# Supplementary material for: Spatial localization of β-unsaturated aldehyde markers in murine diabetic kidney tissue by mass spectrometry imaging
Source: Anal Bioanal Chem. 2022 Jul 26;414(22):6657–70. doi: 10.1007/s00216-022-04229-7 (PMC9411223; doi:10.1007/s00216-022-04229-7)
Supplement: Supplementary file 1 — Supplementary file1 (PDF 1.88 mb) [file 216_2022_4229_MOESM1_ESM.pdf]

Electronic Supplementary Material

for

Spatial Localization of β-unsaturated aldehyde markers in murine diabetic kidney tissue by Mass Spectrometry Imaging

Carla Harkin[a], Karl Smith[b], C. Logan MacKay[c], Tara Moore[d], Simon Brockbank[e], Mark Ruddock and Diego F Cobice[1a]

[a] Mass Spectrometry Centre, Biomedical Sciences Research Institute (BMSRI), School of Biomedical Sciences, Ulster University, Coleraine, Northern Ireland, UK.

[b] National High Magnetic Field Laboratory, Florida State University, Tallahassee, Florida 32310-4005, United State.

[c] Scottish Instrumentation and Research Centre for Advanced Mass Spectrometry (SIRCAMS), EastChem School of Chemistry, University of Edinburgh, Scotland, UK

[d]Genomic Medicine, Biomedical Sciences Research Institute (BMSRI), School of Biomedical Sciences, Ulster University, Coleraine, Northern Ireland, UK

[e] Randox Laboratories Ltd, 55 The Diamond Rd, Crumlin, UK.

[1]Address reprint requests to
Dr Diego Cobice,
Address (a) as above
Tel: +44 (0) 2892604456
Fax: +44(0) 2892604456
Email: d.cobice@ulster.ac.uk

Table of Content

Supplementary Methods **S1**

Supplementary Figures **S2**

Supplementary Results **S3**

1. Supplementary Methods

1.1. LESA-MSI method optimization

To determine the optimal extraction solvent composition, matrix-matched RA standards were analyzed using three alternative ratios of methanol: water: 60:40 % v/v, 70:30 % v/v and 80:20 % v/v, respectively, all prepared with 0.1 % FA (v/v).

A matrix-matched kidney homogenate standard was recovered from -80°C and sectioned using a Leica cryostat (CM 1850 UV; Leica Biosystems, Nußloch, GmbH & CO KG). Three sections were thaw-mounted onto a conductive (ITO-coated) (Bruker Daltonik, Bremen, GmbH & CO. KG) slide and derivatization reagent GT (5ml) (0.1 mg/ml, 0.01 % v/v TFA) was applied. Each of the three sections was analyzed using LESA-MSI using one solvent composition for each section. The slide was analyzed by LESA-MSI using parameters described in **supplementary information, method 1.2**, using the extraction reagents detailed above, one for each section. Best ion production yields for 4-hydroxyhexanal (4-HHE), 4-oxo-2-nonenal (4-ONE) and 4-hydroxynonenal (4-HNE) were achieved using the 70:30 v/v methanol: water ratio (**Fig. S5**).

1.2. LESA-MSI optimized conditions

LESA MSI was performed using a Triversa Nanomate (Advion Biosystems, Inc, Ithaca, NY, USA) coupled to a Thermo Scientific™ TSQ Vantage Triple Stage Quadrupole Mass Spectrometer (Thermo Fisher Scientific, Bremen, GmbH & CO. KG). Spectra were acquired using selected reaction monitoring (SRM) in positive ion mode.

Aspiration was set to 0.9 µl solvent (methanol: water: formic acid 70:30:0.1% v/v). A 0.7 µl volume was dispensed on to the sample surface at a height of 0.4 mm and delayed for 5 s maintaining the liquid micro junction before 1.1 µl was re-aspirated at a height of 0.2 mm and delivered to the nanochip (5 µm internal diameter). Sample delivery time was set to 1 min; nitrogen gas pressure was 0.5 psi, 1.5 kV applied in positive ion mode. The system was cooled to 10°C. Images of kidney sections mounted on slides (600 dpi) were obtained using a scanner (Epson Perfection V330, software version 3.9.2.5 EN, Seiko Epson Corporation, Nagano, Japan) and viewed in LESA Points (Version 1.1.0.0, Advion Biosystems, Inc, Ithaca, NY, USA). A visual

sequence of sample points was created using the image and transferred to ChipSoft Manager (version 8.3.1.1018, Advion Biosystems, Inc, Ithaca, NY, USA). A replicate sequence was generated using Xcalibur (version 3.0.63, Thermo Fisher Scientific, Bremen, GmbH & CO. KG) on the TSQ Vantage system (Thermo Fisher Scientific, Bremen, GmbH & CO. KG). Analytes were detected using selected reaction monitoring (SRM) in positive mode, with a scan width of 1 *m/z*, scan time of 1 s, collision energy 30 V, peak width of 0.70 in Q1 and Q3 (FWHM) with a run time of 1 min (**Table S1**). LESA-MSI images were generated through a series of file type conversions: from .raw thermo file to mzML using ProteoWizard MS Convert GUI tool (ProteoWizard version 3.0.20315.7 Open Source Software, [36,37]) and then to an imzML file using imzML Converter 1.3. This file can then be viewed in Spectral Analysis 1.4 which can present a heat map configuration with ions of interest color graded at relative levels of intensity.

Table S1: LESA-MSI ion transitions

Compound	Precursor *m/z*	Daughter ion *m/z*	Collision Energy (eV)
4-HHE	228.2	110.2	32
4-HNE	268.2	110.2	30
4-ONE	270.2	110.2	35

1.3. LC/MS method gradient

Table S2: Gradient elution conditions for separation of aldehydes by LC/MS from kidney homogenate and serum samples.

Time (min)	% A	% B
0.00	0	100
5.00	0	100
25.00	100	0
30.00	100	0
30.10	0	100
35.00	0	100

1.4. Preliminary (proof-of-concept) RA Experiment

An initial small-scale experiment was performed for screening of potential OTCD reagents, Girard's reagent T (GT) and Dansyl hydrazine (DH), for detection of reactive aldehydes and to investigate signal intensity in diabetic kidney tissues.

Four slides were prepared in the configuration laid out in **Fig. S2(a)**, using one diabetic mouse sacrificed at the third time point (16 weeks) and one lean control. Matrix-matched standards were also included. Optimization of derivatization reagent concentrations and application parameters was adapted from previous works [28,29]. Dansyl hydrazine (DH) (0.1 mg/ml, 0.01 % v/v TFA) and Girard's Reagent T (GT) (0.1 mg/ml, 0.01 % v/v TFA) were applied to slides using a modified 3D printer. No further preparation was required for LESA-MSI. However, CHCA matrix was applied for MALDI-FT-ICR-MSI.

Table S3: Preliminary experiment target analytes and methods applied

Slide No.	Target Analytes	Applied reagents	MSI Method
Prelim A	4-HHE, 4-ONE, 4-HNE	GT + CHCA Matrix	MALDI-FT-ICR-MSI
Prelim B	4-HHE, 4-ONE, 4-HNE	DH + CHCA Matrix	MALDI-FT-ICR-MSI
Prelim C	4-HHE, 4-ONE, 4-HNE	Most effective OTCD reagent from MALDI-MSI only	LESA-MSI

1.5. Untargeted Lipid Analysis

Three slides were prepared in the configuration in **Fig. S2 (b).** Matrix (CHCA) was applied prior to MALDI-FT-ICR-MSI analysis for untargeted lipids. All data was normalized by RMS (root mean square) and post-calibrated calibrated using a matrix (CHCA) peak at 417.04830 *m/z.* MSiReader (Open source, Release 1.02) [40] was employed to isolate discriminative peaks between control and diabetic kidney sections and match them to peaks within a positive ion lipid database (Lipid Maps, https://www.lipidmaps.org) with a mass accuracy of > 5 ppm. Tandem mass spectra was matched against lipid database using Lipid Blast version Full-Release-3, https://doi.org/10.1038/nmeth.2551 (Fiehn Lab, CA, US).

Recurrent matched peaks present in n=3 slides were analyzed further using FlexImaging version 4.1, Bruker Daltonik, Bremen, GmbH & CO. KG) for image generation and SCiLS 2019 cPro (Bruker Daltonik, Bremen, GmbH & CO. KG) for further statistical analysis.

1.6. Histological Staining

Kidney sections were stained using hematoxylin (0.02g/L in ethanol) (Sigma-Aldrich, Dorset, UK) and eosin (0.003g/L in water + 1% Na_2CO_3) dyes as follows: fixed in 20 °C acetone (Honeywell, UK) for 10 min and air-dried. Sections were rehydrated in 70 % v/v ethanol (EtOH) (≥99.8 %, Honeywell, Arlington, UK) (2 min) and tap water (5 min). They were then immersed in hematoxylin dye (6 min), rinsed in water (2 min) and placed in a solution of 10 % v/v acetic acid (Sigma Aldrich, Dorset, UK) in 95 % EtOH (1 min). To enhance the efficacy of the hematoxylin stain, slides were rinsed in water for 15 min in a bluing step. Sections were transferred to eosin dye for 15 s and dipped in water 2-3 times rapidly or until streaking stopped. Stained sections were dehydrated in increasing concentrations of EtOH (50 % - 100 % v/v) for 2 min each and cleared by two changes of xylene (reagent grade, Fisher Scientific, Loughborough, UK) also at 2 min each. Histological mount (Histamount National Diagnostics, Atlanta, US) was applied before sections were completely dry and a glass coverslip was applied. A 600-dpi image was taken of tissues on slides using a scanner (Epson Perfection V330, software version3.9.2.5 EN, Seiko Epson Corporation, Nagano, Japan).

2. Supplementary Figures

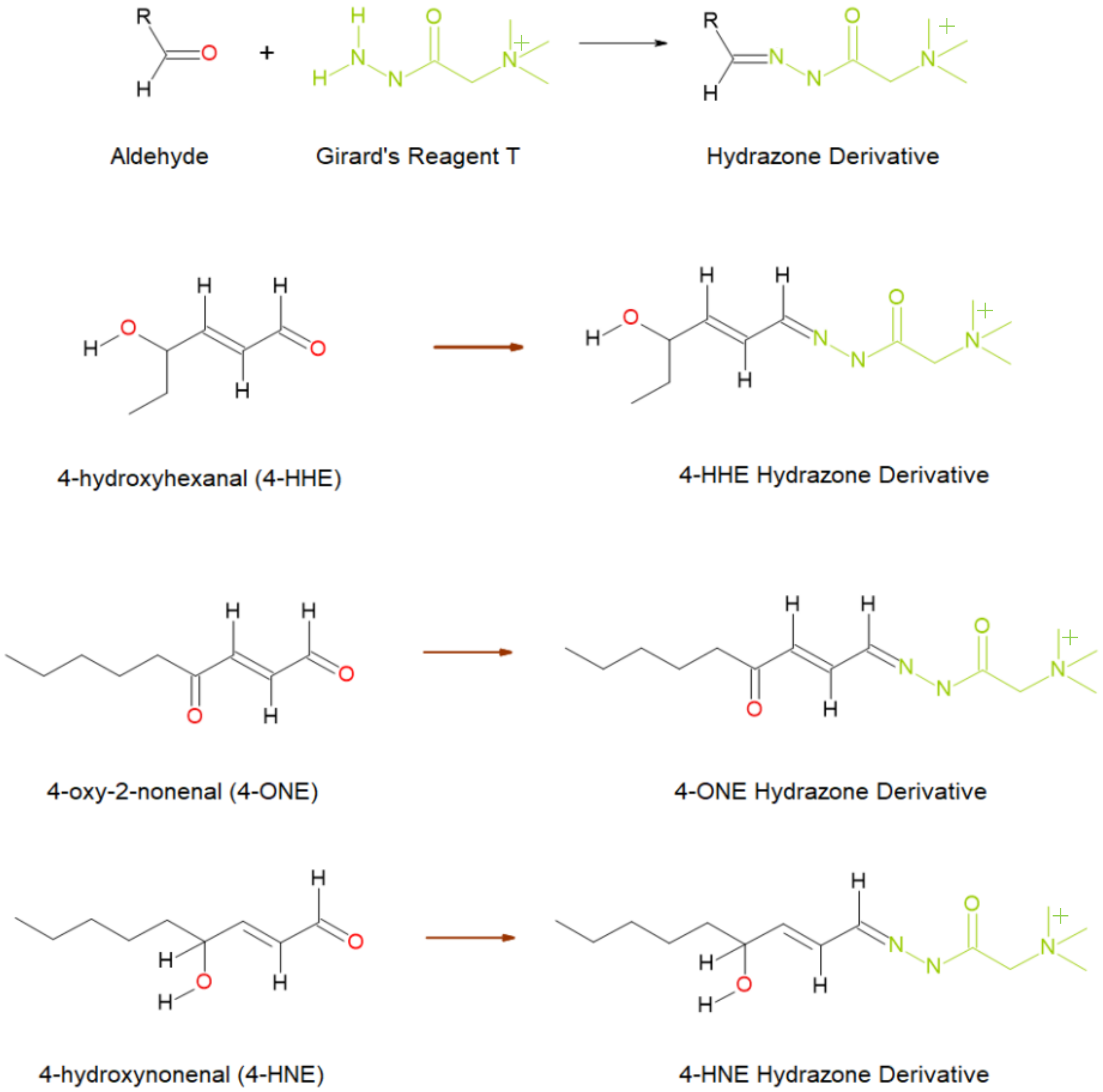

Figure S1: Schematic diagram of the reaction between aldehydes and Girard's reagent T (GT) forming hydrazone derivatives (top). And the hydrazone derivatives formed through reaction with three reactive aldehydes 4-hydroxyhexanal (4-HHE), 4-oxo-2-nonenal (4-ONE) and 4-hydroxynonenal (4-HNE) (below).

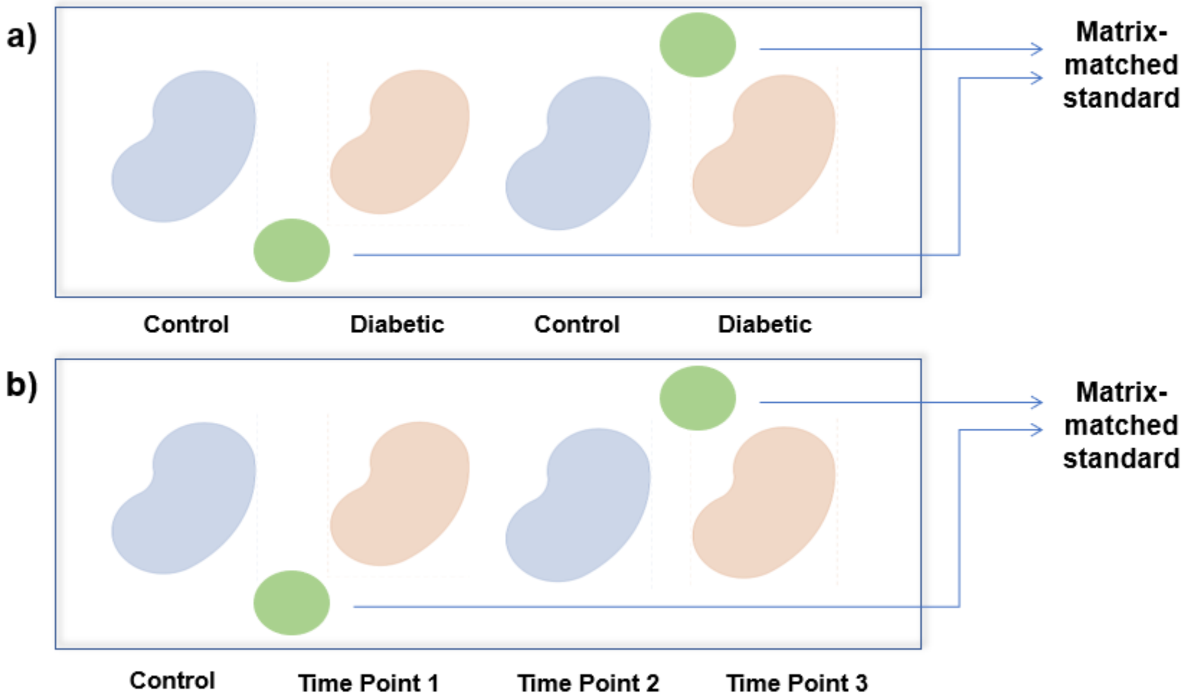

Figure S2: Slide layout for a) preliminary OTCD-MALDI-FT-ICR-MSI screening experiments, where two diabetic tissue sections, two control sections and a matrix-matched standard were analyzed for initial testing and method optimization, and b) Final OTCD-MALDI-FT-ICR-MSI experiments, where one control and diabetic sections from each experimental timepoint (1-3) and a matrix-matched standard were analyzed.

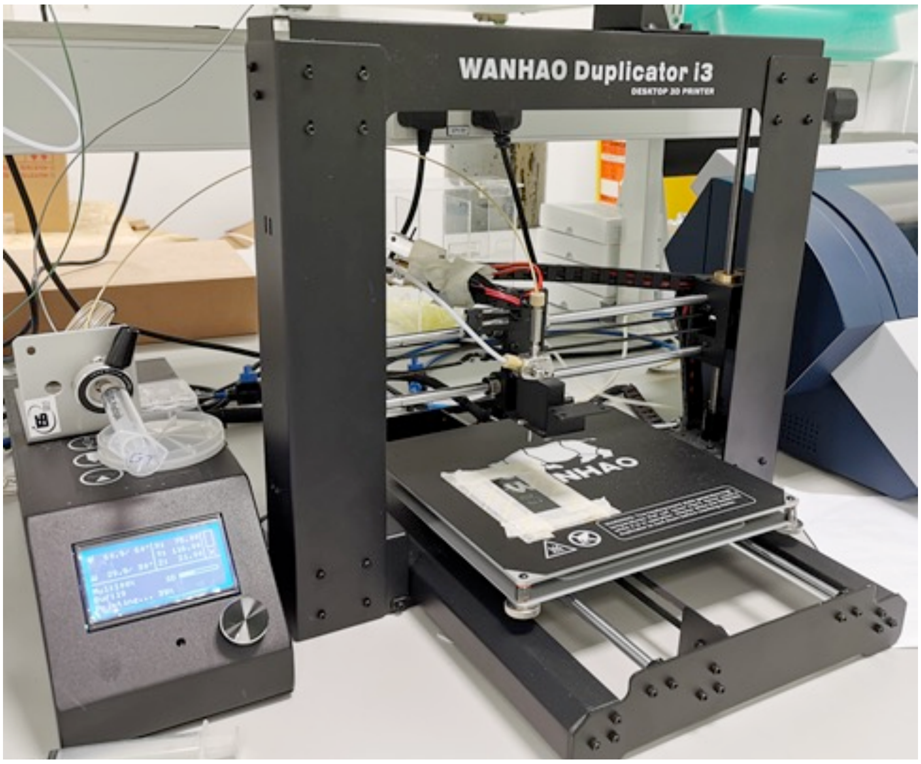

Figure S3: Modified 3D printer used for application of derivatization reagents to tissue sections.

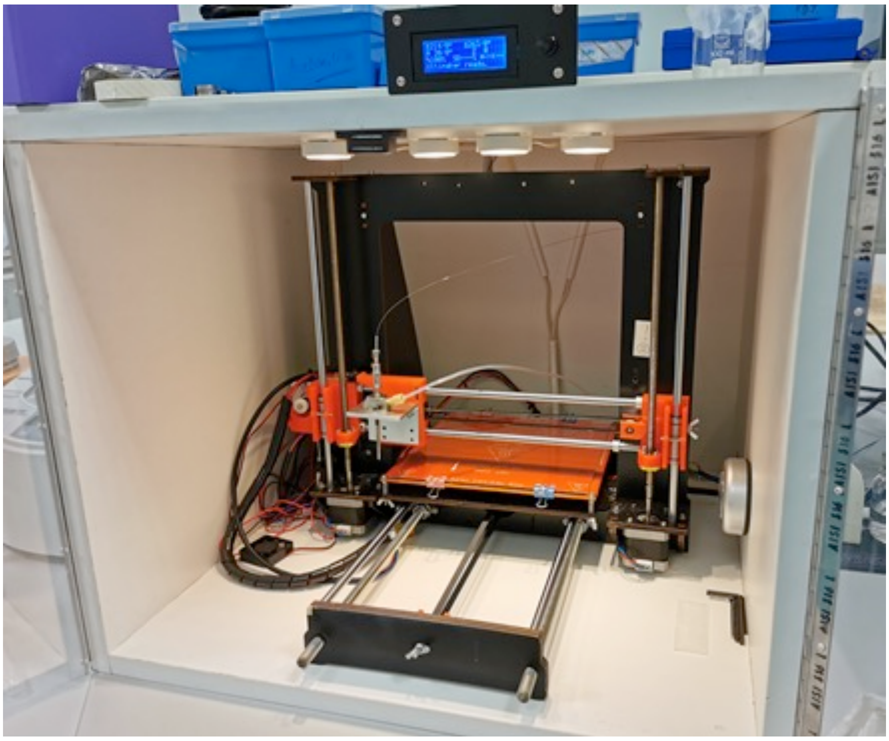

Figure S4: Modified 3D printer used for application of MALDI matrix (CHCA) to tissue sections

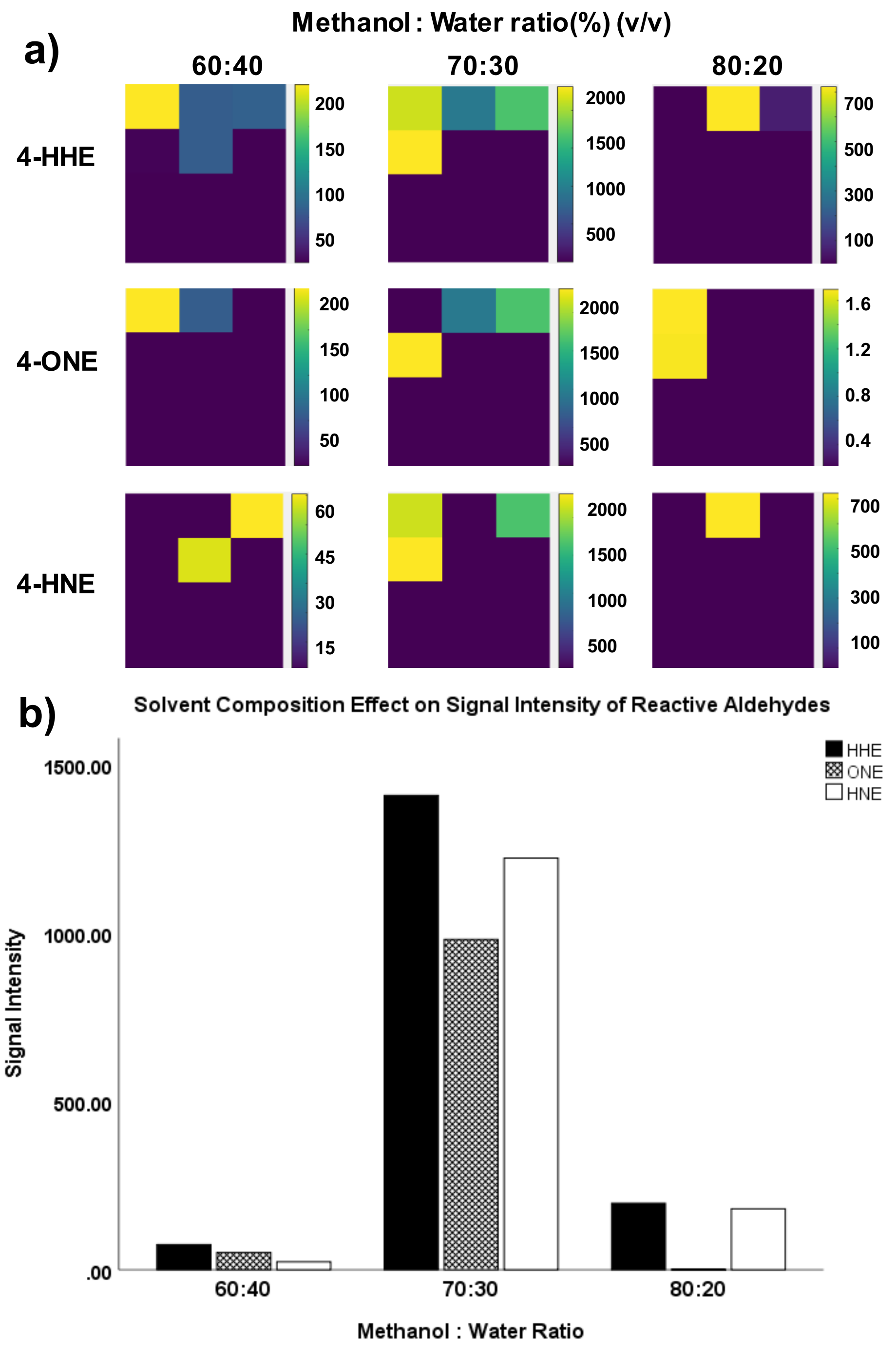

Figure S5: **a)** LESA-MSI images showing the signal intensities (sum of all intensities) and distribution of reactive aldehydes: 4H-HE (parent ion at *m/z* 228.2), 4-HNE (parent ion at *m/z* 268.2) and 4-HNE (parent ion at *m/z* 270.2) from matrix-matched homogenate sections. B) Bar chart displaying signal intensity for each RA at 60:40, 70:30 and 80:20 methanol: water solvent compositions.

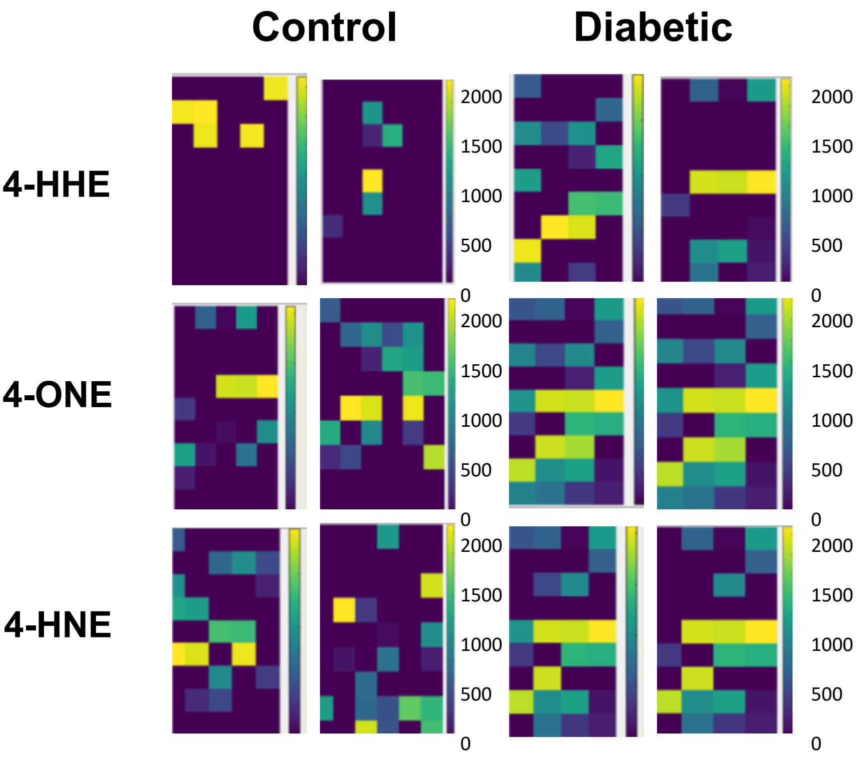

Fig. S6 MSI images from LESA-MSI analysis of preliminary slide with n=2 control (left) and n=2 diabetic (right) adjacent tissues from the same kidney with GT reagent OTCD. Rows top down, MSI images obtained for 4-hydroxyhexanal (4-HHE) (parent ion at *m/z* 228.2), 4-oxo-2-nonenal (4-ONE) (parent ion at *m/z* 268.2) and 4-hydroxynonenal (4-HNE) (parent ion at *m/z* at 270.2). Scale and intensity bars inset.

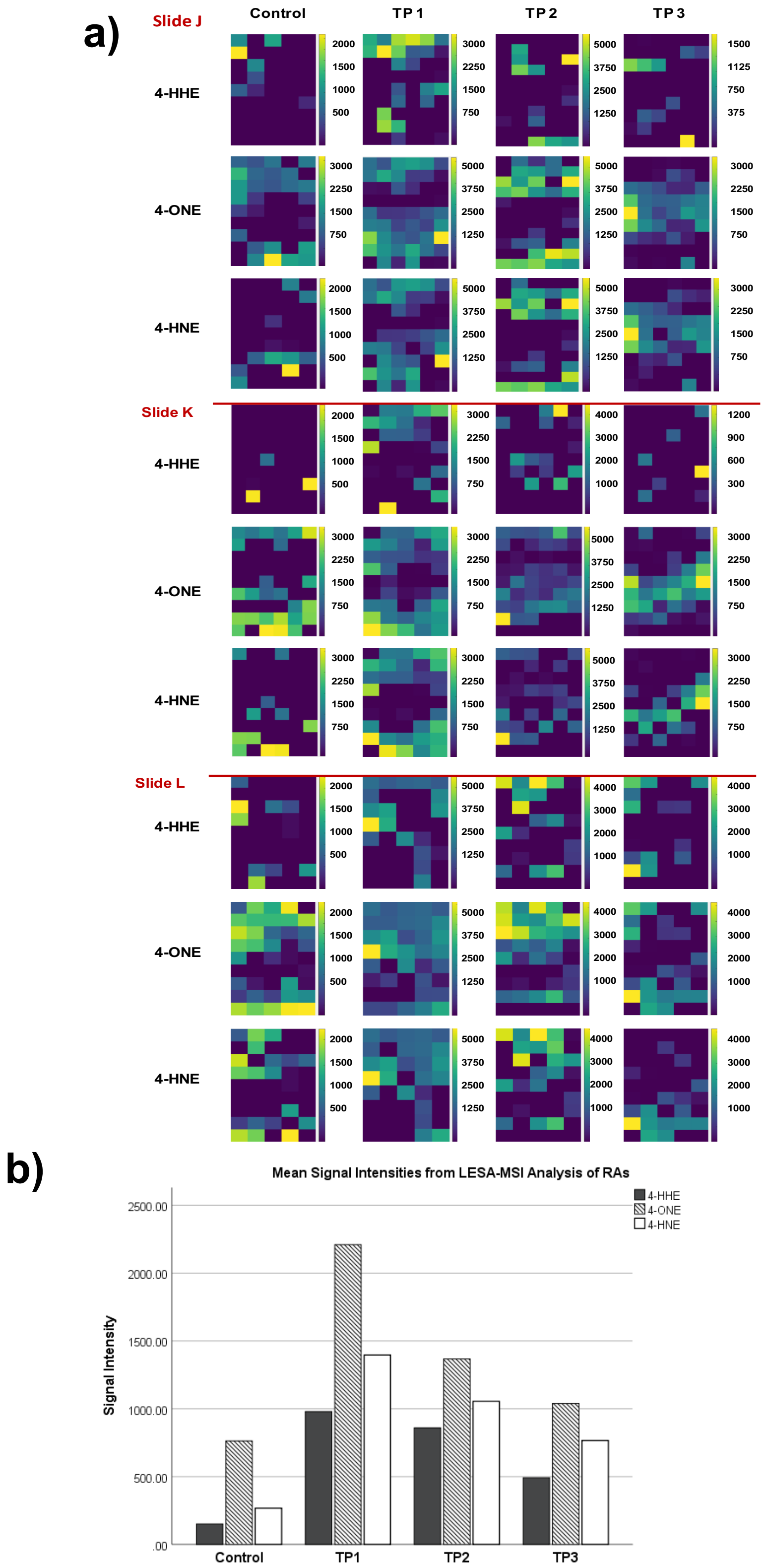

Figure S7: **a)** LESA-MSI images of Slides J, K and L, separated by red line. RAs tested for each aldehyde, rows top-bottom: 4-hydroxyhexanal (4-HHE) (parent ion at *m/z* 228.2), 4-oxo-2-nonenal (4-ONE) (parent ion at *m/z* 268.2) and 4-hydroxynonenal (4-HNE) (parent ion *m/z* at 270.2 *m/z*). Columns l-r: kidney sections, control, and diabetic sections at time point1 (TP1) time point 2 (TP2) and time point 3 (TP3). B) Mean signal intensities of RAs in diabetic kidney sections from LESA-MSI analysis.

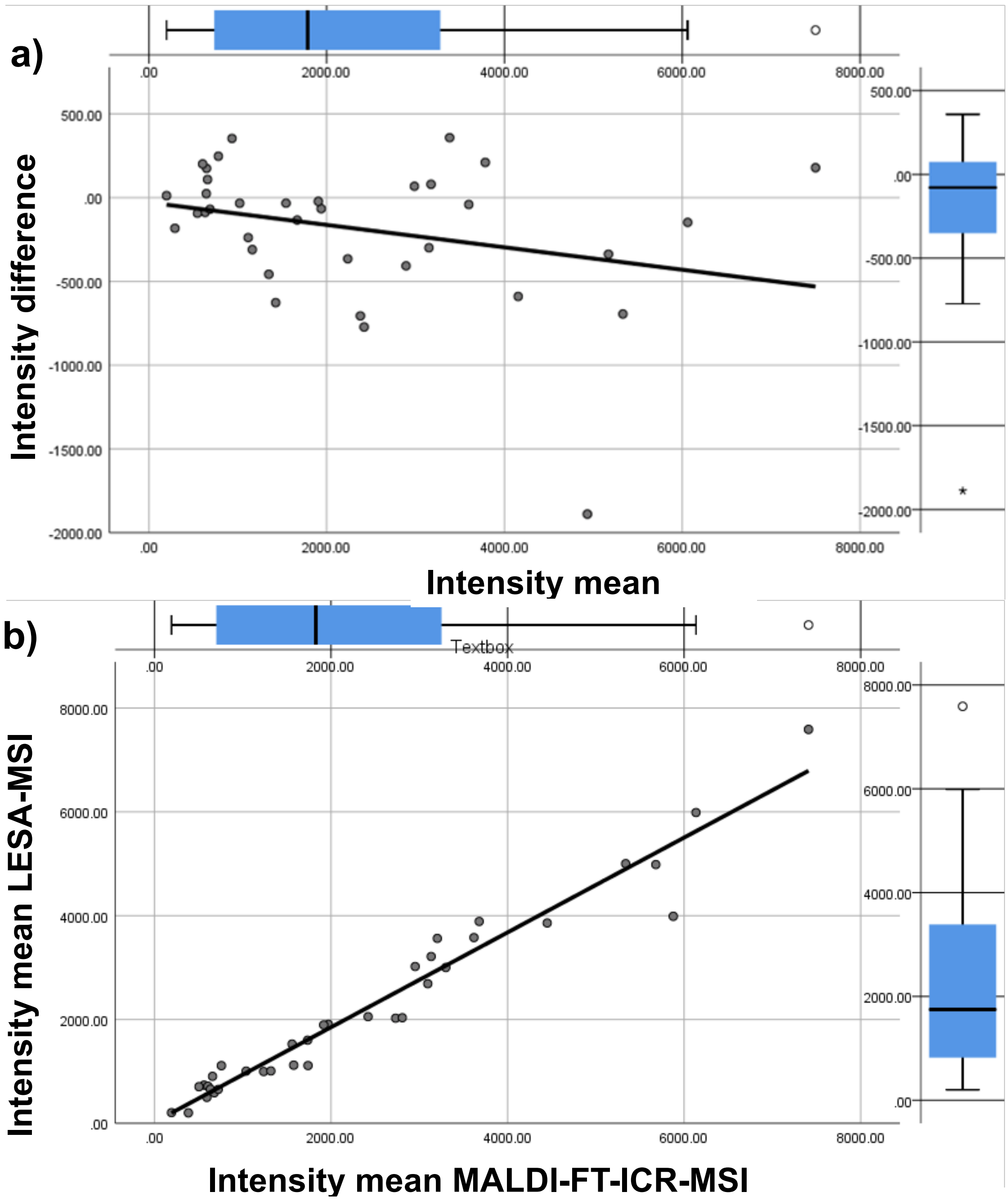

Figure S8: Bland Altman plots showing agreement between LESA –MSI and MALDI-FT-ICR-MSI methods for RA measurement in kidney sections. **a)** Mean versus difference, **b)** Correlation between LESA-MSI (LESA) and MALDI-FTICR-MSI (FT) R^2 value = 0.953.
This was carried out using IBM SPSS Statistics for Windows (Version 25) (SPSS, IBM Analytics, New York, USA).

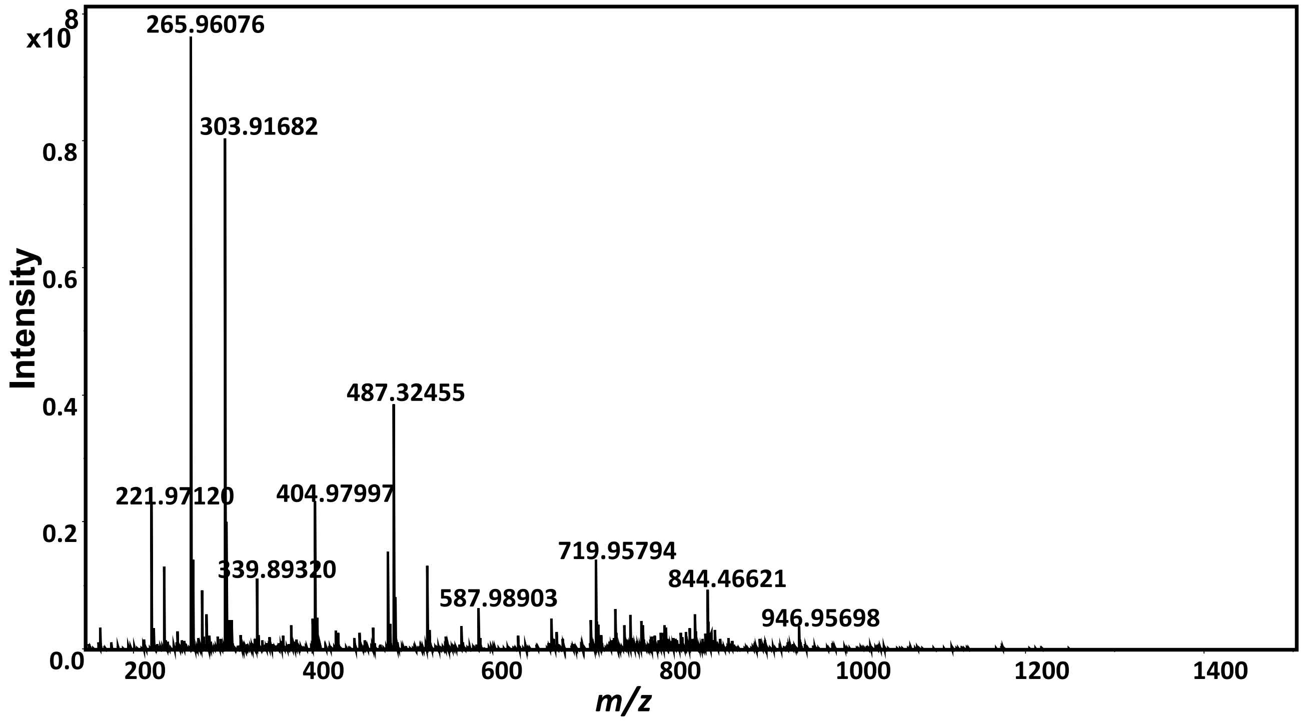

Figure S9: Representative total ion chromatogram (TIC) of a selected region of interest (ROI) showing matrix clusters and endogenous tissue molecular composition. No isobaric interference was observed from either matrix clusters or endogenous molecular components.

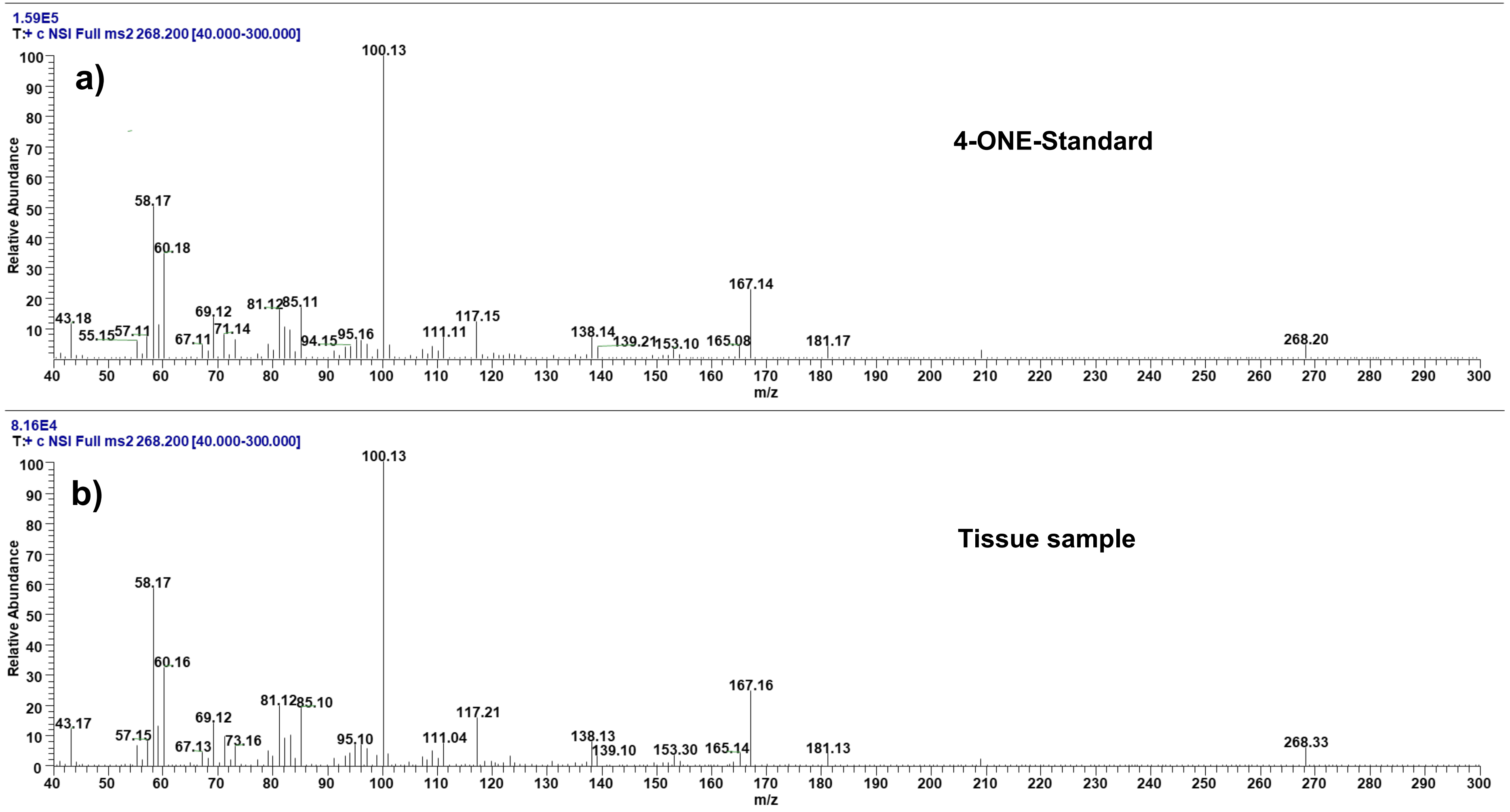

Figure S10: Representative LESA- nESI product ion spectrum of **a)** 4-ONE pure standard at 1 µg/mL **b)** a representative region of interest (ROI) of db/db mouse tissue section at precursor ion *m/z* 268.3

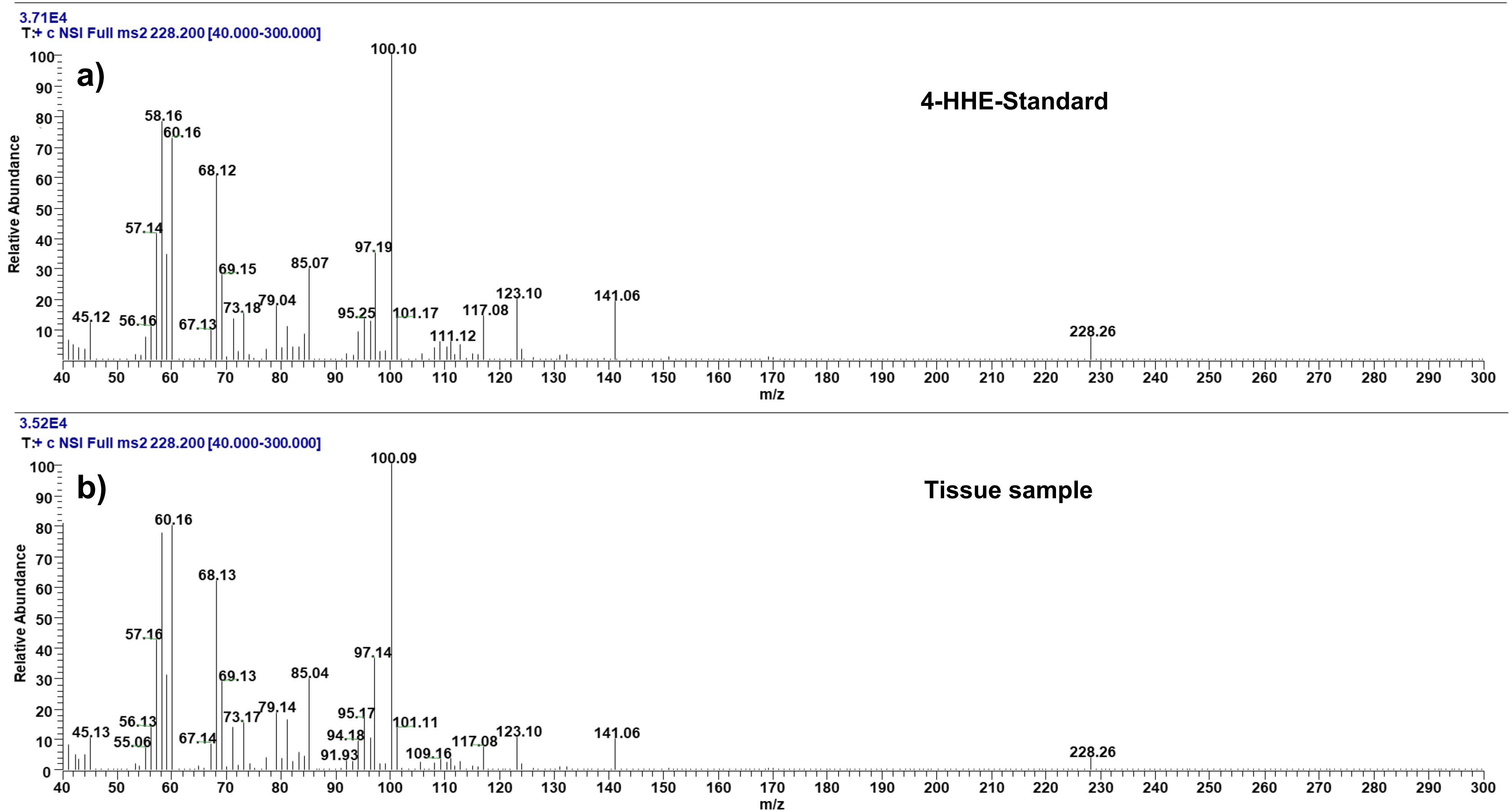

Figure S11: Representative LESA- nESI product ion spectrum of **a)** 4-HHE pure standard at 1 µg/mL **b)** a representative region of interest (ROI) of db/db mouse tissue section at precursor ion *m/z* 228.3

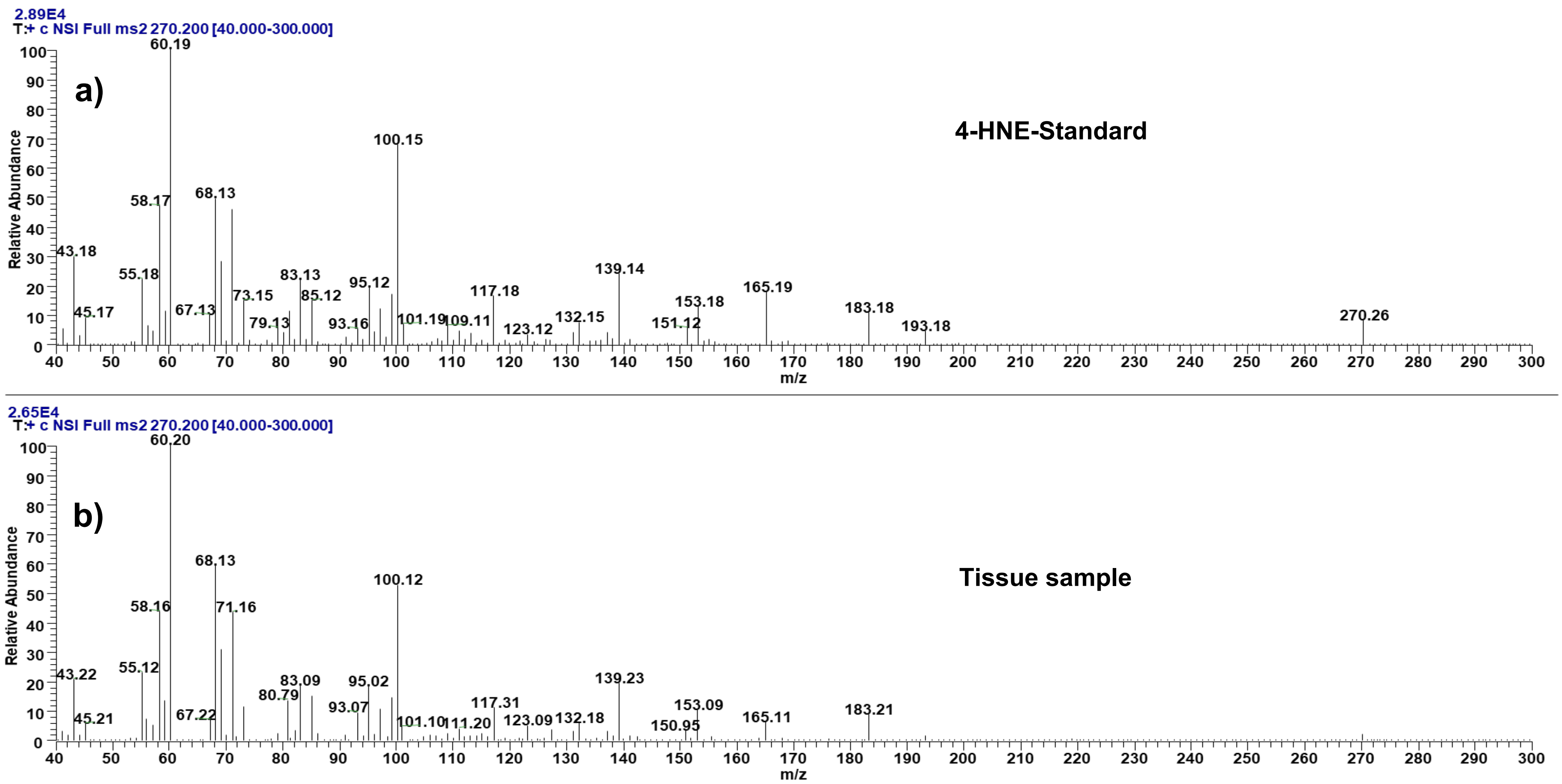

Figure S12: Representative LESA- nESI product ion spectrum of **a)** 4-HNE pure standard at 1 µg/mL **b)** a representative region of interest (ROI) of db/db mouse tissue section at precursor ion *m/z* 270.3

3. Supplementary results

3.1 OTCD-LESA- MSI Analysis

A second, ambient ionization method, LESA- MSI coupled to a triple quadrupole analyzer was used in this study to validate the findings of the MALDI-FT-ICR-MSI analysis. Though the spatial resolution in this case was 1 mm, some localization information was obtained and the trend in signal intensities between groups was matched to MALDI-FT-ICR-MSI results.

During LESA-MSI method development, 70:30% v/v methanol: water was selected as the most effective solvent **(Fig. S5)**. As such, this was used in all further LESA-MSI analysis.

In the preliminary study, following OTCD with GT, LESA-MSI distribution patterns displayed an increase of signal intensity of RAs in diabetic kidney sections compared to a control (non-diabetic/lean) section (**Fig. S6**). Three replicate slides were analyzed to support the larger MALDI-FT-ICR-MSI experiment (**Fig. S7a).** For all RAs tested, the signal intensity in diabetic tissues was greater than control sections. TP1 sections displayed the highest intensity for RAs on all slides, except for 4-ONE on slide L. Mean signal intensities further demonstrate RA detection at higher concentrations at TP1 (**Fig. S7b**). 4-HHE was mainly scattered throughout the section, while 4-ONE and 4-HNE were mostly localized in the cortex at TP1 and TP2 but shifted to medulla at TP3.

3.2 Untargeted Lipid Analysis

Untargeted lipid analysis was conducted on kidney sections to investigate the differences between lipid profiles of diabetic and control tissues. MSiReader (Open source Release 1.02) [40] identified discriminatory lipid peaks between control and diabetic tissues which were matched against a positive ion database with < 5ppm mass accuracy. Flex Imaging was then used to visualize the matched lipid *m/z* intervals. Positive ion-matched peaks are listed in **Table S3.**

Signal for all these lipids was increased in diabetic tissues as observed in the MS images, except at *m/z* 732.55378 which was higher in the control tissue. At TP1, ions at *m/z* 703.57485 and 732.55378 were spatially distributed at a higher intensity

towards the cortex, while ions of *m/z* 723.49352 and 798.54081 were at a higher intensity towards the medulla. At TP2 and TP3, all ions intensities were spatially distributed at a higher intensity in the medulla, except for m/z 732.55378 which is higher in the cortex. Lipid species were further confirmed by tandem MS/MS analysis using Lipid Blast version Full-Release-3, https://doi.org/10.1038/nmeth.2551 (Fiehn Lab, CA, US). The discrimination between control and diabetic tissues demonstrates a change in lipid profile in DN and the possible relationship between lipid peroxidation and RA generation.

Table S3 Main discriminative lipid species identified from control and diabetic kidney tissues with a matched mass accuracy of < 5ppm and MS/MS spectra using Lipid Blast version Full-Release-3, https://doi.org/10.1038/nmeth.2551 (Fiehn Lab, CA, US). Intensity and scale bars for images in table

Ion	**Theoretical**	**Image**				
(m/z)	***(m/z)***	**Control**	**TP1**	**TP2**	**TP3**	**Scale**
703.57485 (SM 18:1_16:0)) $[M+H]^+$	703.5748	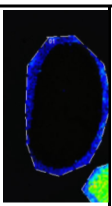	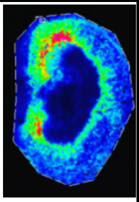	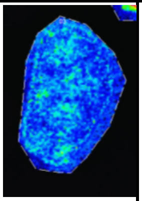	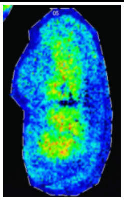	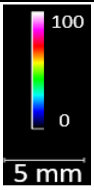
723.49352 PA (18:0_18:2) $[M+Na]^+$	723.4935	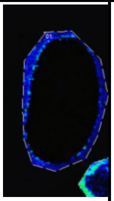	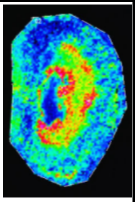	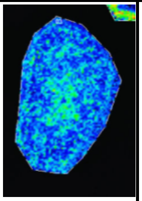	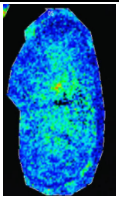	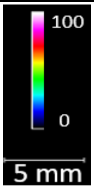
732.55378 PC(18:1_14:0) $[M+H]^+$	732.5538	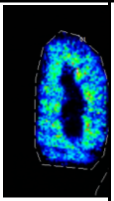	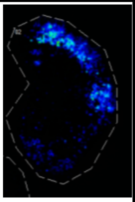	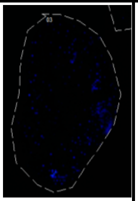	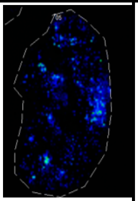	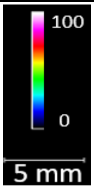
798.54081 PC(18:1_16:0) $[M+K]^+$	798.5410	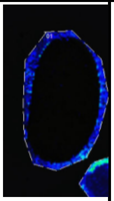	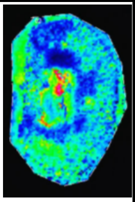	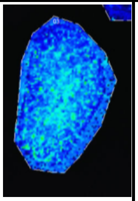	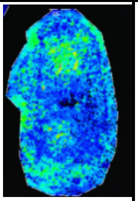	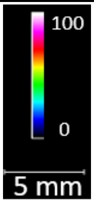

3.3 Partial validation results of the LC/MS method for RAs “based on” m10 bioanalytical method validation guidelines.

Lower Limit of Quantification (LLOQ)

The serum LLOQ for all Ras was 0.5 ng/ml (precision at 10.1-15.6 % and accuracy of 85.2-115.4 %, n=6)

Tissue homogenate LLOQ for all Ras was 1.0 ng/g (precision at 13.4-18.2 % and accuracy of 81.4-112.1 %, n=6).

Selectivity

The selectivity was determined by co-elution assessment analysis of wash, standard 0 (matrix blanks, 0ng/ml) and (LLOQ 0.5 ng/ml and 1.0. ng/g). Neither blank nor solvent washes showed peaks corresponding to RAs.

Matrix effect.

RAs free serum and tissue was used to matrix match. Peak shape, size and quality showed no significant difference between blank and matrix for all RAs in pooled serum and tissue samples. For spiked samples the matrix effect was determined across two concentration levels (1.0 and 25 ng/ml for serum and 10 and 100 ng/g for tissue) with observed accuracies of 85.5-92.3 % (serum) and 91.1-102.3% (tissue) for all RAs and CV% w ranging from 3.4-12.2 % These results show that the matrix effects met the acceptance criteria, and no significant matrix effect was present in the RAs free serum and tissue homogenate

Calibration and dynamic range

Calibration curves were obtained from matrix matching calibration standards (STD 1-7), with the lowest concentration standard at the respective LLOQ (Calibration was assessed by evaluating the deviation of standards from the nominal concentration. All standards passed back calculated accuracy at 100±20% with RAs range 88.0% to 110.0%. A linear regression analysis was performed against ISTD (triplicate) linearity range (0.5-100 ng/ml) for serum and (1-200 ng/g) for tissue, linear regression, and squared correlation coefficient r2 were within 0.994-0.999. Nominals vs back calculated concentrations linear regression analysis for all Ras were within 0.994-0.997. No residual trends were observed.

Accuracy and precision

The accuracy and precision were determined at 1.0 (LQC) and 25 (HQC) ng/ml for serum and 10 (LQC) and 50 ng/g (HQC) for tissue. Sample size was n=6 across two separate analytical runs. Intra-day accuracy ranged between 89.43-99.5% and precision did not exceed 3.2% for all RAs in both serum and tissue. Inter-day accuracy ranged between 93.1%-102.3% and precision did not exceed 6.71%.
Intra analyst precision passed at all 3 analysts with lowest 88.5% accuracy and CV% with the highest result of 3.25%. Inter-analyst accuracy passed at 89.7% and CV% passed at 4.8%.

Carry over

No signals were observed for RAs in a solvent injection followed by HQCs injection showing no significant carry over.

Stability

Autosampler stability was determined for all RAs after sample preparation. Samples were stored at 4˚C for 48 hours after analysis. All results met acceptance criteria after 48 hours for both all RAs with levels against T=0 for all RAs above 85%.
